# Identification of pulmonary arterial hypertension patients with venous or capillary involvement

**DOI:** 10.1093/ajrccm/aamag289

**Published:** 2026-06-11

**Authors:** Adam M Andruska, Alejandro Cruz-Utrilla, Esther J Nossent, Lucas R Celant, Issac Yiqun Yang, Raquel Lyn, Yu Kuang (Julie) Lai, Kristina Kudelko, Yon Sung, Edda Spiekerkoetter, Vinicio De Jesus Perez, Henry Haiwei Guo, Harm Jan Bogaard, Pilar Escribano-Subias, Roham T Zamanian, Andrew J Sweatt

**Affiliations:** Department of Medicine, Pulmonary, Allergy, and Critical Care, Stanford University, Stanford, CA, United States; Vera Moulton Wall Center for Pulmonary Vascular Disease, Stanford University, Stanford, CA, United States; Cardiovascular Institute, Stanford University, Stanford, CA, United States; Pulmonary Hypertension Unit, Department of Cardiology, Hospital Universitario 12 de Octubre, Universidad Complutense de Madrid, Madrid, Spain; Pulmonary Hypertension Core Network, European Reference Network on Rare Respiratory Diseases (ERN-Lung), Brussels, Belgium; Cardiovascular Diseases Program, Centro de Investigación Biomédica en Red de Enfermedades Cardiovasculares (CIBER-CV), Madrid, Spain; Department of Pulmonary Medicine, Amsterdam UMC, Vrije Universiteit Amsterdam, Cardiovascular Sciences Research Institute, Amsterdam, the Netherlands; Department of Pulmonary Medicine, Amsterdam UMC, Vrije Universiteit Amsterdam, Cardiovascular Sciences Research Institute, Amsterdam, the Netherlands; Department of Medical Imaging, University of Toronto, Toronto, Canada; Department of Medical Imaging, Sunnybrook Health Sciences Centre, Toronto, Canada; Department of Medicine, Pulmonary, Allergy, and Critical Care, Stanford University, Stanford, CA, United States; Vera Moulton Wall Center for Pulmonary Vascular Disease, Stanford University, Stanford, CA, United States; Department of Medicine, Pulmonary, Allergy, and Critical Care, Stanford University, Stanford, CA, United States; Vera Moulton Wall Center for Pulmonary Vascular Disease, Stanford University, Stanford, CA, United States; Department of Medicine, Pulmonary, Allergy, and Critical Care, Stanford University, Stanford, CA, United States; Vera Moulton Wall Center for Pulmonary Vascular Disease, Stanford University, Stanford, CA, United States; Department of Medicine, Pulmonary, Allergy, and Critical Care, Stanford University, Stanford, CA, United States; Vera Moulton Wall Center for Pulmonary Vascular Disease, Stanford University, Stanford, CA, United States; Department of Medicine, Pulmonary, Allergy, and Critical Care, Stanford University, Stanford, CA, United States; Vera Moulton Wall Center for Pulmonary Vascular Disease, Stanford University, Stanford, CA, United States; Cardiovascular Institute, Stanford University, Stanford, CA, United States; Department of Medicine, Pulmonary, Allergy, and Critical Care, Stanford University, Stanford, CA, United States; Vera Moulton Wall Center for Pulmonary Vascular Disease, Stanford University, Stanford, CA, United States; Cardiovascular Institute, Stanford University, Stanford, CA, United States; Department of Radiology, Stanford University, Stanford, CA, United States; Department of Pulmonary Medicine, Amsterdam UMC, Vrije Universiteit Amsterdam, Cardiovascular Sciences Research Institute, Amsterdam, the Netherlands; Pulmonary Hypertension Unit, Department of Cardiology, Hospital Universitario 12 de Octubre, Universidad Complutense de Madrid, Madrid, Spain; Pulmonary Hypertension Core Network, European Reference Network on Rare Respiratory Diseases (ERN-Lung), Brussels, Belgium; Cardiovascular Diseases Program, Centro de Investigación Biomédica en Red de Enfermedades Cardiovasculares (CIBER-CV), Madrid, Spain; Department of Medicine, Pulmonary, Allergy, and Critical Care, Stanford University, Stanford, CA, United States; Vera Moulton Wall Center for Pulmonary Vascular Disease, Stanford University, Stanford, CA, United States; Department of Medicine, Pulmonary, Allergy, and Critical Care, Stanford University, Stanford, CA, United States; Vera Moulton Wall Center for Pulmonary Vascular Disease, Stanford University, Stanford, CA, United States; Pulmonary Medicine Section, Veterans Affairs Palo Alto Health Care System, Palo Alto, CA, United States

**Keywords:** pulmonary veno-occlusive disease, pulmonary capillary hemangiomatosis, pulmonary arterial hypertension

## Abstract

**Rationale:**

While therapeutic advances have improved survival for patients with Group 1 pulmonary arterial hypertension (PAH), patients with Group 1.5 “PAH with features of venous/capillary involvement” (formerly pulmonary veno-occlusive disease or pulmonary capillary hemangiomatosis, now collectively termed PVOD/PCH) remain underrecognized, develop serious complications from usual PAH therapy titrations, and suffer high mortality awaiting necessary lung transplant. Identifying PVOD/PCH early before therapy initiation could aid management and expedite transplant referral.

**Objectives:**

We aimed to develop a likelihood score distinguishing PVOD/PCH from other forms of PAH using clinical variables.

**Methods:**

Due to low PVOD/PCH prevalence and no dedicated registries, we leveraged published case-control/case-series studies to assess the ability of several clinical variables to discriminate PVOD/PCH from PAH. From pooled literature-derived data, we performed sensitivity/specificity and simulation-based receiver operating characteristic (ROC) analyses to estimate variable performance. Top-performing variables formed a PVOD/PCH likelihood score, which had its accuracy tested for distinguishing histopathology-confirmed PVOD/PCH cases (*n* = 37) from PAH controls (*n* = 60) in 3 cohorts of transplant-eligible patients from the United States, Spain, and the Netherlands.

**Measurements and Main Results:**

DLCO, 6-minute walk desaturation, Pa_O2_, sex, smoking history, computed tomography (CT) septal line thickening, and CT lymphadenopathy had the highest sensitivity and specificity performance and were incorporated into the PVOD score. Across test cohorts, the score achieved a ROC area under the curve of 0.97 (95% CI, 0.93-1.00) for discriminating PVOD/PCH and it retained accuracy when data were missing.

**Conclusions:**

This score could facilitate early PVOD/PCH identification in incident PAH, potentially helping expedite transplant referral and informing therapy initiation/titration decisions.

At a Glance Commentary **Scientific Knowledge on the Subject:** Group 1.5 “pulmonary arterial hypertension (PAH) with features of venous or capillary involvement” or “PVOD/PCH” is a severe form of PAH characterized by venous occlusion and/or capillary proliferation. Early recognition of PVOD/PCH is critical as patients have difficulty tolerating pulmonary vasodilator therapies, suffer high mortality, and often require lung transplant referral. However, there is no validated tool assessing PVOD/PCH likelihood prior to therapy initiation. Further, a lack of large PVOD/PCH registries complicates deriving and validating a likelihood score with conventional regression or machine learning approaches.

**What This Study Adds to the Field:** To circumvent these challenges, a hybrid approach was used. Existing published literature was used for score derivation, and a new multinational cohort of patients with histologically proven Group 1 PAH was used for validation. The study presents a comparative, literature-derived estimate of the statistical performance of PVOD/PCH discriminating variables. Highest-performing variables form a multiparameter PVOD/PCH likelihood score that is intended for application prior to therapy ­initiation. The score classifies PVOD/PCH from PAH, demonstrates appropriate calibration, and can aid clinicians in phenotyping newly diagnosed Group 1 PAH patients by potentially reducing underrecognition of PVOD/PCH, enabling individualized therapy, or facilitating transplant referral.

## Introduction

Group 1.5 “pulmonary arterial hypertension with features of venous/capillary involvement” is a form of pulmonary arterial hypertension (PAH) characterized by pulmonary venous occlusion and/or capillary proliferation with high mortality.^1-4^ Initially described as the separate entities pulmonary veno-occlusive disease (PVOD) and pulmonary capillary hemangiomatosis (PCH), it is now recognized that PVOD and PCH are clinically indistinguishable pathologic entities, clinically called “PVOD/PCH.”^5^ Diagnosis and management of PVOD/PCH is complicated by a low incidence within PAH populations, a hemodynamic profile indistinguishable from PAH, and poor tolerance to vasodilator therapies.^3^ Because obstructive vascular lesions in PVOD/PCH are located at or distal to the alveolar capillary bed, pulmonary arteriolar vasodilation caused by PAH-directed therapies increases alveolar capillary pressure and can precipitate pulmonary edema and hypoxemia. This precludes treatment escalation, limits right ventricular afterload reduction, and associates with higher mortality.^6^ Because PVOD/PCH is a histopathologic diagnosis and lung biopsy is typically not recommended in Group 1 PAH patients with moderate to severe hemodynamics,^7^ effective PVOD/PCH management first relies on a high clinical suspicion for the disease.^8^ Once a clinical diagnostic threshold for PVOD/PCH is met, referral for lung transplant evaluation is crucial, and physicians are faced with considering treatment plans discordant from current PAH guidelines (eg, avoidance of combinatorial upfront therapy and reduced dosing intensity). Studies suggest that less aggressive vasodilator titrations may improve PVOD/PCH survival and extend time to transplant.^6^^,^^7^^,^^9^ Despite this, PVOD/PCH patients referred for lung transplantation have nearly twice the mortality of PAH patients while completing transplant evaluation or awaiting organ allocation.^10^ There is a critical unmet need to identify PVOD/PCH patients early in their clinical course to afford the best chance of survival to organ allocation.

Clinical suspicion for PVOD/PCH is heightened among Group 1 PAH patients with hypoxemia, severely reduced diffusion capacity, distinct radiographic findings, previous exposures to solvents (eg, trichloroethylene) or chemotherapeutic agents (eg, mitomycin C), bi-allelic mutations in the gene *EIF2AK4*, concomitant connective tissue disease (CTD), or development of pulmonary edema following initiation or escalation of pulmonary vasodilator therapies.^3^^,^^11-17^ However, these criteria require careful interpretation as some are not specific to PVOD/PCH. For example, patients with *EIF2AK4^−/−^* mutational status can have variable presentations and responses to vasodilators, ranging from pulmonary edema at low doses to long-term tolerance.^2^^,^^18^ We are aware of one prior risk assessment tool for PVOD/PCH derived from 19 patients with PVOD and 55 with PAH from a single center.^6^ A pathologic diagnosis was made in 9 of the 19 PVOD patients (47%) and 9 of the 55 PAH patients (16%), indicating that the underlying diagnosis was predominately based on expert clinical assessment. The predictor carrying the greatest weight for PVOD risk was “pulmonary edema due to vasodilators,” which may not be known at the time of initial diagnosis. Also, “defect in perfusion lung scan” is a risk prediction variable that has been shown by other groups to have variable ability to discriminate PVOD/PCH from PAH.^19^

Although PVOD/PCH is rare, variable prevalence rates reported in the literature suggest it goes underrecognized. This underrecognition in part relates to clinicians lacking a standard approach to signal PVOD/PCH early in disease presentation. Identifying patients with PVOD/PCH prior to vasodilator initiation could improve outcomes by informing the need for less aggressive vasodilator titrations and early transplant referral.

Our primary study objective was to develop a tool to assess the likelihood of PVOD/PCH among transplant-eligible PAH patients before therapy initiation. However, we recognized that derivation of such a tool would demand a nontraditional study approach given the low number of PVOD/PCH patients in single-institution datasets and the lack of accessible multicenter registries containing matched data from PVOD/PCH and PAH patients with histologic diagnoses. We hypothesized that existing published data could be leveraged to build a simulated PVOD/PCH patient registry from which a likelihood tool could be derived. To enhance the clinical practicality and applicability of this tool, we prioritized readily available noninvasive clinical variables. Finally, upon deriving a PVOD/PCH likelihood score from published data, we tested its performance in assessing the gold-standard outcome of histologic PVOD/PCH. The study population is explicitly comprised of World Symposium on Pulmonary Hypertension (WSPH) Group 1 patients with either known vascular pathology (PVOD or PCH for cases and plexiform arteriopathy for PAH controls) or a known, PAH-specific genetic mutation. The study population does not include patients with Group 2 to 5 pulmonary hypertension, the Group 1 PAH “lung phenotype,” or mixed pulmonary hypertension phenotypes. Some of the results of these studies have been previously reported in the form of an abstract.^20^

## Methods

**Figure 1:**
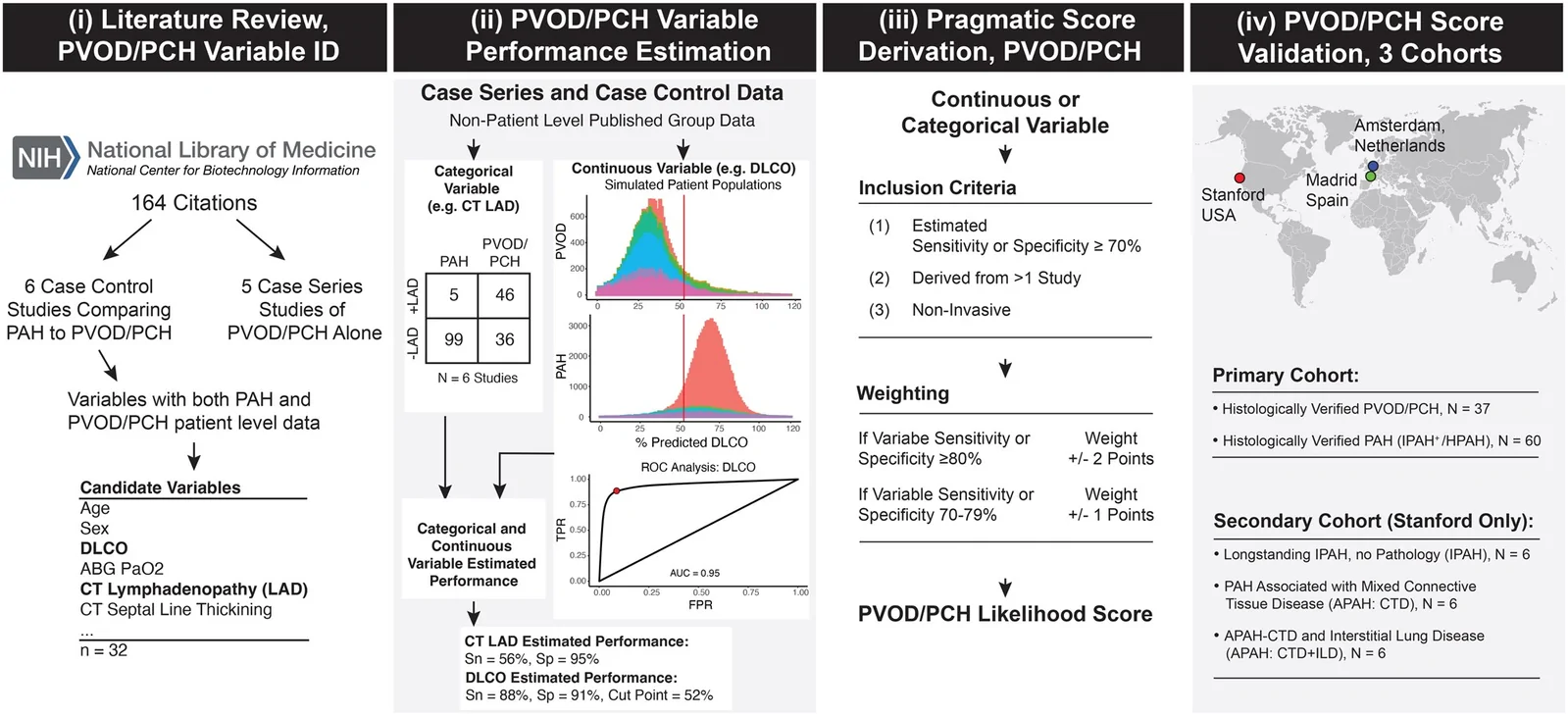
Graphical abstract of study methodology detailing (i) variable identification from the literature; (ii) derivation of estimated sensitivity and specificity from published non-patient-level data to assess variable performance; (iii) the development of a pragmatic PVOD/PCH likelihood score based on the highest-performing, literature-derived variables; and (iv) validation of the pragmatic likelihood score in 3 independent multinational cohorts. Abbreviations: ABG, arterial blood gas; APAH, associated pulmonary arterial hypertension; CT, computed tomography; CTD, connective tissue disease; FPR, false-positive rate; HPAH, heritable pulmonary arterial hypertension with a pathogenic *BMPR2* or *TBX4* mutation; IPAH, non–pathologically verified idiopathic pulmonary arterial hypertension; IPAH^+^, histologically verified idiopathic pulmonary arterial hypertension; ILD, interstitial lung disease; LAD, lymphadenopathy; PAH, Group 1 pulmonary arterial hypertension; PVOD/PCH, Group 1.5 pulmonary arterial hypertension with features of venous/capillary involvement; Sn, sensitivity; Sp, specificity; TPR, true-positive rate.

The overall study methodology is outlined in Figure 1. We (1) reviewed published PVOD/PCH literature and identified variables potentially distinguishing PVOD/PCH from PAH, (2) quantitatively evaluated each variable’s PVOD/PCH discrimination accuracy, (3) developed a pragmatic PVOD/PCH likelihood assessment score using the top-performing variables, and (4) tested the score’s PVOD/PCH discrimination accuracy on the outcome of histopathology-verified PVOD/PCH versus PAH in 3 cohorts from Stanford University, United States; the Hospital Universitario 12 de Octubre, Madrid, Spain; and Amsterdam UMC, the Netherlands.

Starting with 164 references, we narrowed to 6 case-control^3^^,^^6^^,^^12^^,^^13^^,^^19^^,^^21^ studies that published direct comparisons of clinical data for PVOD/PCH and PAH patients for 32 clinical variables. Five case-series studies^2^^,^^4^^,^^9^^,^^22^^,^^23^ had data specific to PVOD/PCH patients alone and were used in sensitivity and specificity estimates.

Performance of the 32 variables was assessed by estimating their sensitivity and specificity for PVOD/PCH using data published in case-control and case-series studies. Categorical variable sensitivity and specificity were directly calculated from 2 × 2 contingency tables after tabulating patient numbers across all studies (Table S1). Continuous variable performance was calculated by generating simulated patient data using truncated normal distributions defined by the published mean and standard deviation (or median and range).^24^ Simulated patient distributions were pooled for each variable and study, and receiver operating characteristic curve (ROCC) analyses^25^ of the summed population estimated area under the curve (AUC) performance, sensitivity, and specificity of the variable as a function of cut point. A single cut point for each continuous variable maximizing sensitivity and specificity (Youden’s point^26^) was used. Sensitivity and specificity were estimated if at least one study contained matched PAH and PVOD/PCH cohort data. Twenty-six of 32 variables used data from multiple case-control studies. The number of patients used to estimate variable sensitivity and specificity equaled the number of patients published in the literature for categorical variables and was proportional to the size of published study populations for categorical variables (Table S1 and Figure S2). PVOD/PCH-only case-series studies were included in variable estimation to increase sample size and represent patient diversity across cohorts. For variables with AUC >0.9 (percent predicted Dl_CO_ and arterial blood gas [ABG] Pa_O2_), 2 cut points were used to maximize sensitivity and specificity (Figure S2).

Because our derivation data from published studies describe cohort-level attributes and not patient-level data, we could not employ regression, Bayesian, or machine learning techniques to assess variable interactions and perform traditional model development. Instead, a pragmatic approach was required to convert estimated variable performances to a likelihood score with clinical utility. Our heuristic scheme assigned positive points if a patient met or surpassed ROCC-defined PVOD/PCH sensitivity cut points for a continuous variable (eg, Dl_CO_ <50%) or, for categorical variables, had the condition (eg, computed tomography [CT] lymphadenopathy). Several rules guided score development: We (1) included variables with performance estimated by more than one study, (2) used a lower bound cutoff (sensitivity or specificity of >70%) for variable inclusion, and (3) gave variables with superior performance (sensitivity or specificity ≥80%) more weight (Figure 1-iii). Negative points were assigned for patient values meeting the ROCC specificity cut point for PVOD/PCH (continuous) or absence of the condition (categorical). Certain classic predisposing factors for PVOD/PCH are not included in the score due to a lack of data allowing specificity and sensitivity estimation or a lack of available data in test and validation cohorts (Table S3).

Likelihood score performance was evaluated on the outcome of histologically determined PVOD/PCH. To calculate likelihood scores for subjects in the validation cohorts, we collected clinical data available closest to the time of their initial PAH diagnosis by right heart catheterization, prior to therapy initiation. Details of this pathologic assessment of patient lung samples are provided in the Online Supplement. In brief, explanted lung tissue from patients undergoing transplant or deceased patients undergoing autopsy was stained with standard hematoxylin and eosin, Elastica van Gieson, CD34, and reticulin stains. PVOD/PCH or PAH status was adjudicated by the clinical pathologist at each institution. Primary patient cohorts were grouped as histologically verified idiopathic PAH (termed IPAH^+^), heritable PAH with a pathogenic *BMPR2* or *TBX4* mutation (HPAH), and histologically verified PVOD or PCH (PVOD/PCH). For all primary cohorts, we excluded patients with either significant restriction and radiographic fibrosis or significant obstructive lung disease (defined as FEV_1_/FVC <0.70 and FEV_1_ <0.60).

Secondary, non–pathologically verified idiopathic PAH (IPAH) and CTD-associated PAH without (APAH-CTD) and with (APAH-CTD-ILD) interstitial lung disease cohorts were included from Stanford USA data.

Subjects with excessive missing data were excluded according to criteria established a priori (Table S4). Residual missing data were handled by both zero and multivariate predictive mean matching imputation. STrengthening the Reporting of OBservational studies in Epidemiology (STROBE) diagrams for all cohorts are shown in Figure S3. Details pertaining to patient inclusion, exclusion, secondary cohorts, and statistical analysis are provided in the Online Supplement.

## Results

### Published data enables ranking of variables distinguishing PVOD/PCH from PAH

From 6 PVOD/PCH case-control studies, we identified 7 categorical and 25 continuous variables that reported data for PAH and PVOD/PCH patients (Figure 1). For categorical variables, the presence of lymphadenopathy on CT exhibited the greatest sensitivity and specificity (Table 1). Aggregate and study-specific sensitivity and specificity are shown in Table S1. In general, categorical CT variables—lymphadenopathy, septal line thickening, and ground glass opacity (GGO)—displayed high specificity but lower sensitivity, indicating utility for ruling in PVOD/PCH (Table 1).

**Table 1:** Literature-derived performance estimates of candidate PVOD/PCH distinguishing variables.

Characteristic	No. of Studies	ROCC	Youden’s point analysis			
		AUC	Cut point	Unit	Sensitivity	Specificity
Continuous variables						
** Golde score on BAL^a^**	1	0.96	24		0.92	0.97
** Hemosiderin-laden Mø BAL^a^**	1	0.95	14	%	0.87	0.95
**DlCO, % predicted^b^ ^,^ ^c^**	8	0.95	52	%/Pred	0.90	0.91
** 6MWT lowest Sp_O2_ ^c^**	3	0.92	87	%	0.86	0.91
** 6MWT Delta Sp_O2_ ^a^**	1	0.89	7.9	%	0.79	0.85
** Resting Sp_O2_ ^a^**	1	0.88	94.5	%	0.79	0.81
** ABG Pa_O2_ ^b^ ^,^ ^c^**	6	0.84	67	mm Hg	0.77	0.75
**DlCO/VA, % predicted^a^**	4	0.80	53	%/Pred	0.74	0.73
** Pack-years of smoking^c^**	2	0.79	17	Pack-years	0.70	0.73
** MAP on RHC^a^**	1	0.73	83	mm Hg	0.59	0.75
** 6MWT distance**	8	0.65	300	m	0.61	0.62
** Age^a^**	12	0.63	56	Years	0.54	0.75
** TLC**	5	0.61	95	%/Pred	0.56	0.62
** BMI**	4	0.61	27	kg/m^2^	0.51	0.71
** FEV_1_/FVC**	2	0.61	80		0.54	0.64
** Cardiac output, RHC**	4	0.60	4.4	L/min	0.49	0.75
** PVR, RHC**	7	0.57	9.4	WU	0.48	0.68
** PAWP, RHC**	7	0.56	8.3	mm Hg	0.55	0.55
** FEV_1_**	8	0.56	88	%/Pred	0.46	0.74
** ABG Pa_CO2_**	6	0.56	31	mm Hg	0.49	0.61
** mPAP, RHC**	9	0.56	49	mm Hg	0.44	0.72
** Cardiac index, RHC**	8	0.53	2.6	L/(kg·min)	0.60	0.46
** PVR index, RHC**	1	0.52	22.5	WU/kg	0.44	0.64
** FVC**	5	0.51	90	%/Pred	0.42	0.73
** RAP on RHC**	4	0.51	9	mm Hg	0.50	0.52
**Categorical variables**						
** Lymphadenopathy on CT^c^**	6				0.56	0.95
** Septal line thickening on CT^c^**	5				0.49	0.83
** Centrilobular GGO on CT^c^**	5				0.56	0.79
** Male sex^c^ ^,^ ^d^**	12				0.67	0.70
** Pericardial effusion on CT**	3				0.30	0.46
** Never smoked**	5				0.29	0.30
** Pleural effusion on CT**	3				0.22	0.90

Abbreviations: 6MWT, 6-minute walk test; ABG, arterial blood gas; AUC, area under the curve; BMI, body mass index; CT, computed tomography; GGO, ground glass opacity; Mø, macrophage; MAP, mean arterial pressure; mPAP, mean pulmonary artery pressure; PAWP, pulmonary artery wedge pressure; PVOD/PCH, group 1.5 pulmonary arterial hypertension with features of venous/capillary involvement; PVR, pulmonary vascular resistance; RAP, right atrial pressure; RHC, right heart catheterization; ROCC, receiver operating characteristic curve; VA, alveolar volume.

a Excluded from score development; see text.

b Due to performance, 2 cutoffs are chosen to maximize sensitivity and specificity (Table S2).

c Used in risk prediction score.

d Applicable for sporadic cases not associated with chemotherapy, solvent exposures, or connective tissue disease.

Continuous variable performance analysis is shown in Figure S1. A single study showed that hemosiderin-laden macrophages on BAL had the highest sensitivity and specificity for PVOD/PCH. Percent predicted Dl_CO_, oxygen saturation data on 6-minute walk test (6MWT), and oxygen levels on the ABG (Table 1) had high sensitivity and specificity across multiple studies. Simulated distributions, ROCC analysis, sensitivity, specificity, and cut points are given on a per-study and aggregate basis in Figure S2. We observed that some variables, such as 6MWT distance, had a wide range of ROC AUC (0.5-0.9) across studies, which did not support their use as a consistent PVOD/PCH-distinguishing variable. Conversely, variables like percent predicted Dl_CO_ demonstrated AUC uniformity across studies in combination with a high sensitivity and specificity to support utility for PVOD/PCH discrimination.

### Top-performing variables inform a PVOD/PCH likelihood score

**Figure 2:**
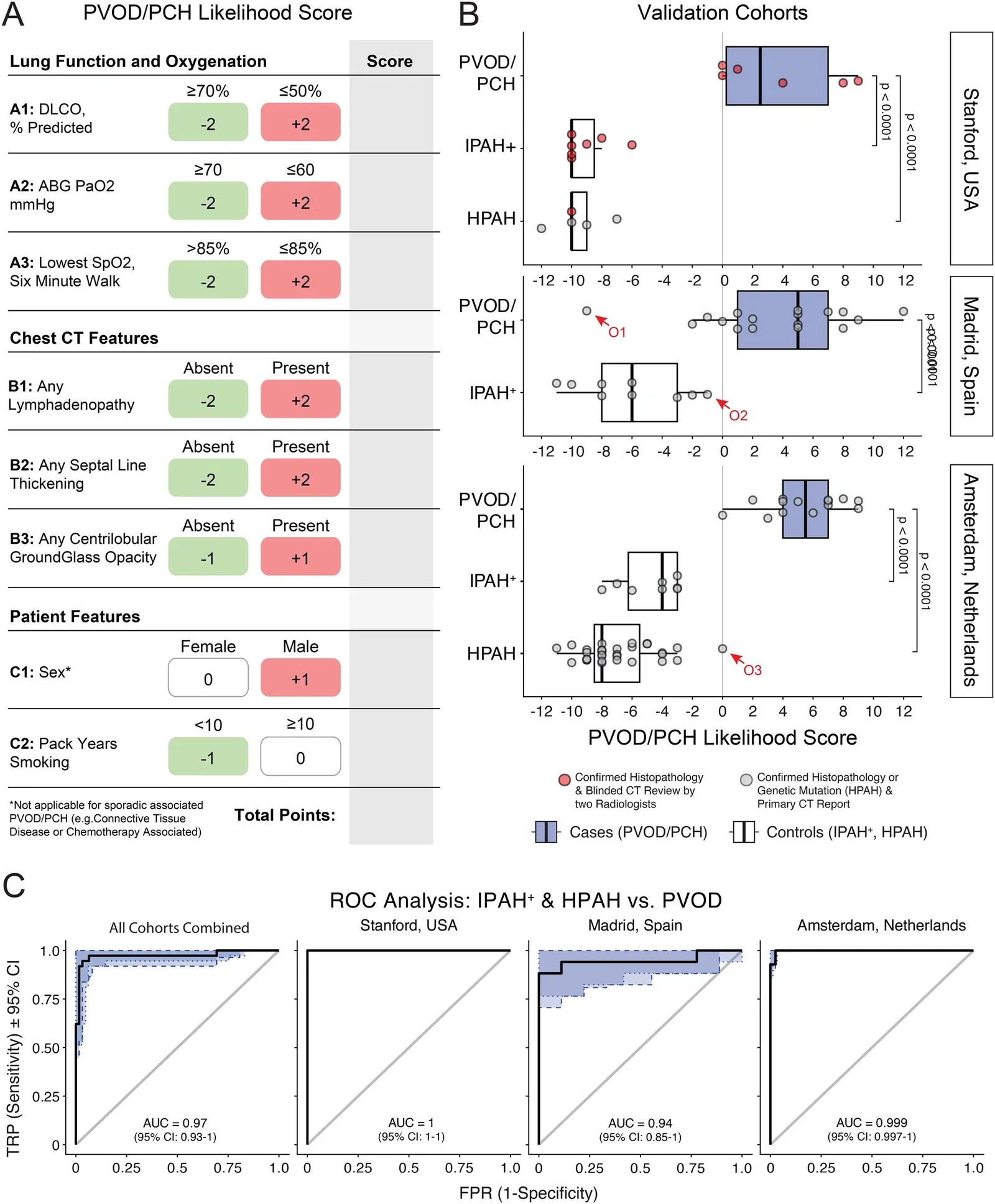
(A) The literature-derived pragmatic PVOD/PCH likelihood score. Assessment occurs in 3 domains: (1) lung function and oxygenation, (2) chest CT features, and (3) patient features. The final score is the sum of all positive and negative points. Note that for patients with the potential for associated forms of PVOD/PCH (eg, mixed connective tissue disease, chemotherapy exposure, or occupational exposures), sex is not considered a risk factor and should be omitted. Additionally, a detailed occupational or exposure history should be undertaken. If the CT scan is missing at the time of assessment, data from all (B) and (C) subcomponents are required to apply the score. If the CT scan is present, data from at least 2 of 3 variables in (B) and one of 2 variables in (C) are sufficient to apply the score. For missing data, zero imputation may be done and generates a similar outcome compared to computational missing data imputation methods (see Figure S6B). (B) The PVOD/PCH likelihood score distinguishes PVOD/PCH from different PAH classes in 3 cohorts: Stanford, United States; Madrid, Spain; and Amsterdam, Netherlands. O1-O3 indicates outliers described further in the text and Table S5. For the Stanford and Netherlands validation cohort, a one-way ANOVA was performed investigating PVOD/PCH likelihood score versus patient class. *P* values are generated from a post hoc Tukey test. For the Spain validation cohort, *P* values are calculated using a Welch 2-sample t-test. Gray dots indicate cases where chest CT features were derived from the earliest available CT report and the diagnosis was adjudicated by either pathology or, in the case of HPAH, a known genetic mutation or familial history (see description below). Red dots indicate that chest CT features were assessed by 2 independent radiologists blinded to the underlying diagnosis and the diagnosis was adjudicated by either pathology or, in the case of HPAH, a known genetic mutation or familial history. (C) ROC curve analysis of all cohorts combined and each independent cohort. For these analyses, cases are PVOD/PCH patients (shaded boxes in B above), and controls are IPAH^+^ and HPAH patients combined (open boxes in B above). In each plot, AUC ± bootstrap 95% CI is indicated. Blue lines and shading indicate 95% bootstrap confidence bounds around the sensitivity (dashed lines) and specificity (dotted lines). Abbreviations: ABG, arterial blood gas; AUC, area under the curve; CT, computed tomography; FPR, false-positive rate; HPAH, heritable pulmonary arterial hypertension with established mutation (*BMPR2* or *TBX4*) or an established familial pulmonary arterial hypertension history; IPAH^+^, patients with histologically verified idiopathic pulmonary arterial hypertension undergoing blinded radiologist review of CT scans or independent review of original CT report; PAH, pulmonary arterial hypertension; PVOD/PCH, patients with pathologically validated Group 1.5 pulmonary arterial hypertension with features of venous/capillary involvement undergoing blinded radiologist review of CT scans or independent review of original CT report; ROC, receiver operating characteristic; TPR, true-positive rate.

Among candidate variables analyzed from the literature that were noninvasive and had data available from multiple studies, those with the best PVOD/PCH discriminatory accuracy included percent predicted Dl_CO_, lowest saturation achieved on 6MWT, ABG Pa_O2_, Dl_CO_/alveolar volume, pack-years of smoking, lymphadenopathy on CT, septal line thickening on CT, centrilobular GGO on CT, and male sex. A PVOD/PCH likelihood score containing these top-performing variables was constructed according to methods described above (Figure 2A).

### Cohort demographics, missing data, and exclusion

STROBE flowcharts for all cohorts are detailed in Figure S3. In total, 60, 54, and 501 incident Group 1 PAH cases from Stanford, Madrid, and Amsterdam were screened. Of the cases with verified histopathology (PVOD/PCH, IPAH^+^) or genetic analysis (HPAH), 10 Stanford, 18 Spain, and 28 Netherlands cohort patients were excluded due to missing data. Distributions of missingness by variable are shown in Figure S6A. A total of 37 PVOD, 24 IPAH^+^, and 36 HPAH patients were included in the study. Seven Spain and 2 Netherlands PVOD/PCH patients had *EIF2AK4*^−/−^ status. Baseline demographics are shown in Table 2. Stanford and Netherlands PVOD/PCH patients were older than those in the Spain PVOD/PCH cohort (*P* <.01). Netherlands PVOD/PCH patients exhibited higher incidence of CT centrilobular GGO compared to their Stanford and Spain counterparts (*P* <.01). Spanish IPAH^+^ patients were younger than those in other cohorts (*P* = .01). Stanford IPAH^+^ patients had lower cardiac output (*P* = .01) and higher percent predicted Dl_CO_ (*P* = .02). Notably, centrilobular GGO was observed more commonly in IPAH^+^ than PVOD/PCH patients in the Stanford cohort and at similar rates between groups in the Spain cohort. Baseline demographics for secondary Stanford cohorts (eg, CTD-associated PAH and non–histologically verified PAH) are presented in Table S2.

**Table 2:** Cohort baseline characteristics.

**Variable**	**Etiology**	Cohorts									Between-Cohort
		Stanford, USA			Madrid, Spain			Amsterdam, Netherlands			
		*N*	Mean	(SD)	*N*	Mean	(SD)	*N*	Mean	(SD)	*P* value
**Age, y **	PVOD/PCH	6	**54.3**	**(14.1)**	17	**26.4**	**(4.2)**	14	**47.6**	**(15.8)**	**<.001**
	IPAH^+^	7	**35.3**	**(13.9)**	9	**24.2**	**(6.4)**	8	**43.4**	**(17.1)**	**.021**
	HPAH	5	39.6	(9.0)				31	41.6	(14.1)	.76
**RHC mPAP, mm Hg**	PVOD/PCH	6	54.0	(11.0)	17	46.8	(15.3)	14	48.9	(14.5)	.59
	IPAH^+^	7	56.9	(11.5)	9	57.0	(14.6)	8	64.1	(26.4)	.68
	HPAH	5	60.2	(20.0)				31	55.6	(14.4)	.53
**RHC CO, L/min**	PVOD/PCH	6	3.6	(1.0)	17	5.3	(1.2)	14	4.8	(1.8)	.08
	IPAH^+^	7	**2.7**	**(0.8)**	9	**4.1**	**(1.0)**	8	**4.4**	**(0.9)**	**.0090**
	HPAH	5	4.4	(1.2)				31	4.5	(1.3)	.94
**RHC PVR, WU **	PVOD/PCH	6	13.3	(3.2)	17	10.3	(3.5)	14	9.6	(6.2)	.27
	IPAH^+^	7	**16.5**	**(6.8)**	9	**6.2**	**(3.1)**	8	**14.6**	**(8.4)**	**.0065**
	HPAH	5	13.0	(7.8)				31	11.0	(4.5)	.42
**DlCO, % Decimal % predicted**	PVOD/PCH	6	0.47	(0.10)	17	0.42	(0.23)	14	0.39	(0.17)	.77
	IPAH^+^	7	**0.84**	**(0.13)**	9	**0.67**	**(0.10)**	8	**0.64**	**(0.11)**	**.0055**
	HPAH	5	**1.03**	**(0.08)**				31	**0.77**	**(0.16)**	**.0015**
**FVC, % Decimal % predicted**	PVOD/PCH	6	0.75	(0.11)	17	0.92	(0.19)	14	0.98	(0.18)	.05
	IPAH^+^	7	1.01	(0.16)	9	0.84	(0.14)	8	0.96	(0.20)	.15
	HPAH	5	0.86	(0.06)				31	1.03	(0.22)	.094
**FEV_1_, % Decimal % predicted**	PVOD/PCH	6	0.77	(0.11)	17	0.86	(0.17)	14	0.87	(0.15)	.44
	IPAH^+^	7	0.95	(0.12)	9	0.79	(0.15)	8	0.82	(0.16)	.090
	HPAH	5	0.82	(0.10)				31	0.96	(0.18)	.11
**Pack-years of smoking**	PVOD/PCH	6	11.0	(16.1)	17	15.3	(19.5)	14	8.6	(12.7)	.59
	IPAH^+^	7	4.0	(7.7)	9	4.0	(7.0)	8	9.5	(11.2)	.41
	HPAH	5	1.9	(4.2)				31	2.3	(6.7)	.89
**BMI, kg/m^2^ **	PVOD/PCH	6	27.0	(6.2)	17	26.4	(4.2)	14	26.6	(4.8)	.97
	IPAH^+^	7	27.4	(6.9)	9	24.2	(6.4)	8	23.6	(11.6)	.67
	HPAH	5	**33.6**	**(5.8)**				31	**26.9**	**(5.6)**	**.020**
**Pa_O2_ on ABG, mm Hg**	PVOD/PCH	6	62	(11)	17	58	(12)	14	64	(21)	1
	IPAH^+^	7	78	(9)	9	70	(15)	8	66	(13)	.24
	HPAH	5	73	(11)				31	58	(27)	1
**6MWT: lowest Sp_O2_, %**	PVOD/PCH	6	89	(7)	17	79	(8)	14	81	(6)	1
	IPAH^+^	7	94	(4)	9	86	(9)	8	88	(7)	.18
	HPAH	5	95	(3)				31	92	(4)	.093
**6MWT: starting Sp_O2_, %**	PVOD/PCH	6	93	(4)	17	91	(4)	14	91	(4)	1
	IPAH^+^	7	96	(3)	9	94	(4)	8	94	(2)	.70
	HPAH	5	97	(3)				31	97	(3)	.83
**Male sex, %**	PVOD/PCH	6	**33%**		17	**59%**		14	**14%**		**.027**
	IPAH^+^	7	0%		9	11%		8	38%		.16
	HPAH	5	40%					31	35%		1
**Ever smoked, %**	PVOD/PCH	6	83%		17	63%		14	46%		.32
	IPAH^+^	7	29%		9	44%		8	63%		.46
	HPAH	5	20%					31	23%		1
**Chest CT GGO, % **	PVOD/PCH	6	**33%**		17	**35%**		14	**93%**		**.0017**
	IPAH^+^	7	**100%**		9	**33%**		8	**38%**		**.012**
	HPAH	5	40%					31	17%		.27
**Chest CT SLT, %**	PVOD/PCH	6	83%		17	47%		14	86%		.06
	IPAH^+^	7	0%		9	11%		8	13%		1
	HPAH	5	0%					31	7%		1
**Chest CT LAD, %**	PVOD/PCH	6	100%		17	53%		14	79%		.089
	IPAH^+^	7	14%		9	11%		8	0%		.74
	HPAH	5	0%					31	3%		1

Data are presented as mean (standard deviation) or percentage. Between-cohort *P* values are calculated by one-way ANOVA for continuous variables or by Fisher exact test for count data. Bold values indicate significant between-cohort comparisons.

Abbreviations: 6MWT, 6-minute walk test; ABG, arterial blood gas; BMI, body mass index; CO, cardiac output; CT, computed tomography; GGO, centrilobular ground glass opacity; HPAH, heritable pulmonary arterial hypertension with a pathogenic *BMPR2* or *TBX4* mutation; IPAH^+^, histologically verified idiopathic pulmonary arterial hypertension; LAD, lymphadenopathy; mPAP, mean pulmonary artery pressure; PVOD/PCH, group 1.5 pulmonary arterial hypertension with features of venous/capillary involvement; PVR, pulmonary vascular resistance; RHC, right heart catheterization; SLT, septal line thickening; WU, Wood units.

### Likelihood score distinguishes PVOD/PCH from PAH

The likelihood score distinguished PVOD/PCH from IPAH^+^ and HPAH in the Stanford cohort (Figure 2B). The score performed similarly in the Spain and Netherlands validation cohorts (Figure 2B). In a pooled analysis of all 3 cohorts, with IPAH^+^ and HPAH combined into a larger “PAH” category, the likelihood score distinguished PVOD/PCH from PAH with an AUC of 0.97 (95% CI, 0.93-1.00) (Figure 2C). PVOD/PCH likelihood score distributions were similar across cohorts and patient classes regardless of the missing data imputation method applied (zero imputation vs sophisticated predictive mean matching^27^; Figure S6B).

### Sensitivity analysis demonstrates similar performance across different score methods

Four alternative models were used to investigate the effect of varying variable inclusion thresholds and weighting approaches on test performance (Table S6). Across all 4 methods, discriminatory performance remained consistently high (Figure S8; ROCC AUC range, 0.959-0.974). These findings suggest that the observed discrimination performance of the PVOD/PCH likelihood score is driven by the underlying variables chosen rather than by any single weighting scheme.

### Impact of individual variable predictors within the PVOD/PCH score

The composite score significantly outperformed any single component. Standalone performance varied across individual predictors, with the best discriminatory value observed for Dl_CO_, CT lymphadenopathy, and septal line thickening, and the worst discrimination for sex, CT GGO, and smoking history (Figure S5B). Nonetheless, the composite score maintained strong performance with omissions of each single predictor (AUC remained >0.95) (Figure S5A) or the omission of all 3 CT imaging parameters (AUC decreased to 0.92 [95% CI, 0.87-0.98]).

### Failure analysis

In Figure 2B, 3 patients (O1-O3) had outlier PVOD/PCH likelihood scores compared to their respective groupings. Patient O1 is a PVOD/PCH patient with likelihood score −9. Patients O2 and O3 are IPAH^+^ and HPAH patients with likelihood scores of −1 and 0, respectively. The PVOD/PCH outlier lacked both features of hypoxemia and classic radiographic features of PVOD/PCH. The IPAH^+^ and HPAH outliers both had a history of smoking with significant hypoxemia and borderline Dl_CO_ reduction (65% and 52%) relative to other IPAH^+^ and HPAH subjects. Full details of these outliers are provided in Table S5.

### Calibration, score performance, and thresholds

**Figure 3:**
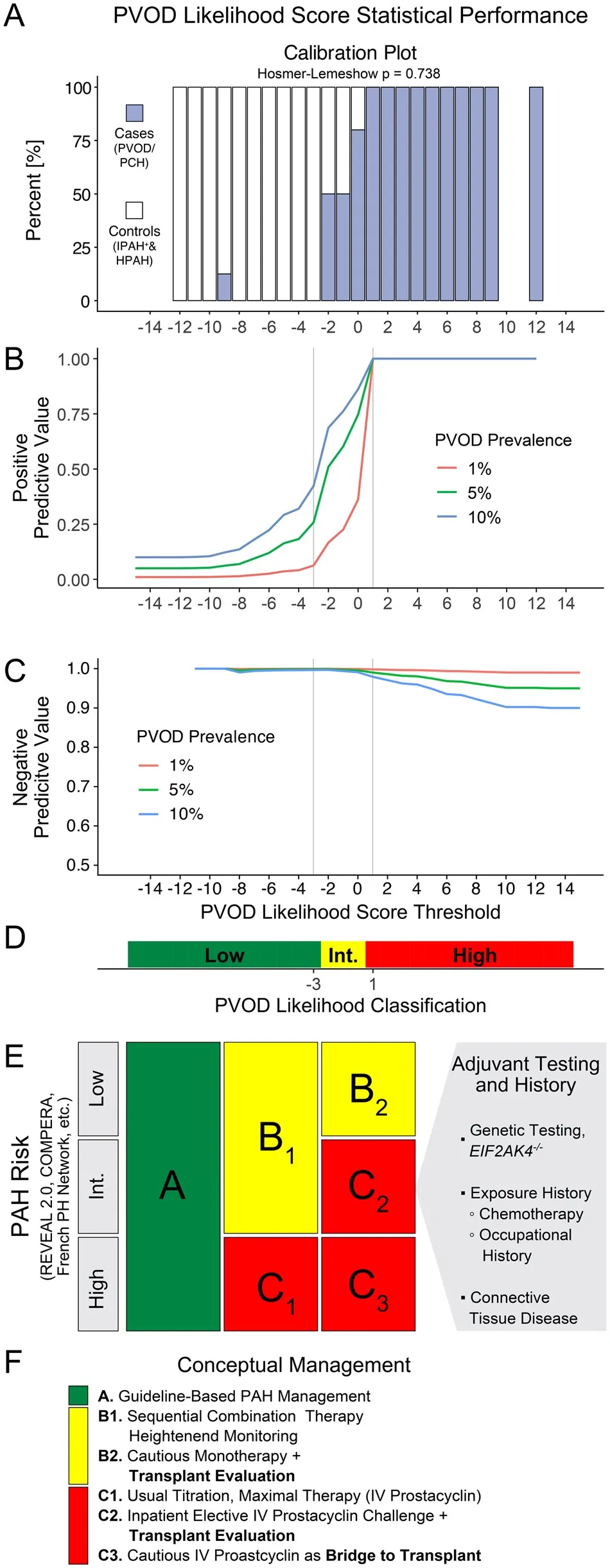
Calibration plots, NPV, and PPV of the pragmatic PVOD/PCH likelihood score with possible incorporation into clinical management. (A) Simplified calibration plot showing the proportion of PVOD/PCH and PAH (IPAH^+^ and HPAH combined) cases as a function of PVOD/PCH likelihood score. The Hosmer–Lemeshow statistic (χ^2^ = 5.18, *P* = .7378) assessed calibration, or agreement between predicated and observed PVOD/PCH probabilities. In the case of the null hypothesis being true (large *P* value), there is a good fit between observed PVOD/PCH rates and predicted probabilities across probability deciles. Details of the generalized linear model used to calculate the Hosmer–Lemeshow *P* value are given in the Supplemental Methods. Graphical illustration of the Hosmer–Lemeshow results is shown in Figure S7. (B and C) PPV and NPV values derived from the sensitivity and specificity calculated at each score value (eg, sensitivity at score −2 is the number of PVOD/PCH patients with scores at or above −2 divided by total PVOD/PCH patients; specificity at score −2 is number of PAH patients with score below −2 divided by total PAH patients). Because PPV and NPV are dependent on prevalence, 3 values of prevalence (1%, 5%, and 10%) are used. Vertical black lines at scores −3 and +1 indicate potential cut points for breaking the populations into low-, intermediate-, and high-likelihood PVOD/PCH categories. (D and E) A conceptual framework for integrating the PVOD/PCH likelihood score into current PAH management, assisted by established PVOD/PCH risk factors such as occupational exposure history, connective tissue disease history, and genetic analysis. The PVOD/PCH likelihood score can assess the likelihood of PVOD/PCH independently to PAH risk, as illustrated by the color coded 3 × 3 table. (F) Depending on the overall PVOD/PCH likelihood and PAH risk, patients can be stratified into different treatment pipelines that justify heightened monitoring, more cautious inpatient vasodilator titrations, or expedited lung transplant referral. Note that the study population for the PVOD/PCH likelihood score includes classic World Symposium on Pulmonary Hypertension Group 1 PAH patients as the control population, while excluding Group 2, 3, 4, or 5 patients. Additionally, the score does not include PAH patients with comorbidities (including the so-called “lung phenotype”). Application of the PVOD/PCH score to non–Group 1 PAH patients or Group 1 PAH patients with comorbidities would be expected to result in increased false positives, eg, high PVOD/PCH likelihood. Abbreviations: HPAH, heritable pulmonary arterial hypertension with a pathogenic *BMPR2* or *TBX4* mutation; IPAH^+^, histologically verified idiopathic pulmonary arterial hypertension; NPV, negative predictive value; PAH, Group 1 pulmonary arterial hypertension; PPV, positive predictive value; PVOD/PCH, Group 1.5 pulmonary arterial hypertension with features of venous/capillary involvement.

To assess agreement between predicted and observed PVOD/PCH probabilities, calibration analysis was performed (Figure S7). A simplified calibration plot in Figure 3A shows PVOD/PCH and PAH probabilities as a function of PVOD/PCH likelihood score. Hosmer–Lemeshow analysis found a goodness of fit statistic of 5.78 and *P* value of .672, indicating a good fit between the risk prediction score and the binary outcome of PVOD/PCH and PAH across probability deciles. Calculated positive and negative predictive values are presented in Figure 3B and C. We choose cut points of −3 to stratify between low and intermediate likelihood PVOD/PCH and +1 to stratify between intermediate- and high-likelihood PVOD/PCH (Figure 3D). This strategy results in 2.7% of PVOD/PCH patients (outlier patient O1) and 95.1% of PAH patients in the 3 cohorts being placed in the low-likelihood PVOD/PCH category, and 16.2% of PVOD/PCH patients and 4.9% of PAH patients grouped as intermediate-likelihood PVOD/PCH. Finally, 0% of PAH patients and 81.1% of PVOD/PCH patients are placed the high-likelihood PVOD/PCH category. Percentages of patients allocated to each likelihood class as a function of score cut point are shown in Figure S9.

## Discussion

Although current guidelines indicate that PVOD/PCH should be considered in group 1 PAH patients with disproportionately low Dl_CO_ and characteristic radiographic abnormalities,^7^ to the best of our knowledge there exists no validated tool guiding clinicians in this determination prior to therapy initiation. A recent study of clinically diagnosed PVOD/PCH patients found that PAH risk prediction models may underestimate mortality, with the best-performing European Society of Cardiology/European Respiratory Society 4-strata model having a c-statistic of 0.64 on index assessment.^4^ A study of histologically confirmed PVOD/PCH patients also found that patients stratified to both intermediate- and high-risk groups had similarly high rates of death or lung transplantation.^28^ Thus, traditional PAH risk scores may under report the mortality risk of group 1.5 PVOD/PCH patients. Here, we present the first comparative estimate of the statistical performance of PVOD/PCH discriminating variables from literature-derived data. This enables a multiparameter PVOD/PCH likelihood score designed for early application prior to therapy initiation, validated in independent cohorts of histologically confirmed PAH and PVOD/PCH. This tool can aid clinicians challenged with phenotyping newly diagnosed group 1 PAH, potentially reducing underrecognition of PVOD/PCH. Further, early knowledge of PVOD/PCH likelihood prior to therapy start could individualize PAH therapy for patients, reduce time to transplant referral, or potentially improve chances for organ allocation.

The approach for developing the PVOD/PCH likelihood score was intentionally pragmatic and shaped by the constraints of studying a rare, underrecognized disease. Lacking existing multicenter repository with sufficient patient-level data from pathologically confirmed PVOD/PCH cases and adjudicated PAH controls, we leveraged published case-control cohort statistics to identify consistently discriminating variables and construct a clinically interpretable score. Inherent statistical limitations of this approach must be acknowledged, including reliance on simulated data, assumptions of normality, inability to model correlations or interactions, and information loss from dichotomization at thresholds. Conventional regression, Bayesian, or machine-learning approaches were infeasible and would have imposed unverifiable assumptions on joint distributions and covariance structures. To address concerns relating to heuristic score construction, we performed sensitivity analysis of alternative model strategies showing stable discrimination across methods (Table S6, Figure S8), indicating that performance is driven by underlying variables rather than a specific scoring scheme. Importantly, generalizability was evaluated in 3 independent multinational datasets of pathologically or genetically confirmed diagnoses, limiting circular inference and center-specific overfitting. While our score development framework did not permit formal optimism correction or coefficient calibration, it enabled rigorous external validation on ground-truth data and addressed the challenge of predictive model development in a rare disease context. Prospective study of broader, systematically adjudicated PAH populations would be required to calibrate the model and determine clinical benefit.

We found that centrilobular GGO on CT scan in isolation has a lower performance for discriminating PVOD/PCH both in our aggregate literature analysis and in our patient cohorts (Table 1, Figure S5B). Independent, blinded, and randomized CT review found that 100% of pathologically verified IPAH^+^ patients in the Stanford cohort had centrilobular GGO on CT versus 33% of PVOD patients (Table 2). Low discriminatory value for centrilobular GGO is seen with CT reports in the Madrid (45% of IPAH^+^ vs 41% of PVOD/PCH) and Netherlands (33% of IPAH^+^ vs 93% of PVOD/PCH) cohorts. However, interlobular septal thickening and adenopathy maintain high discriminatory power for PVOD/PCH. A limitation is that we could not control the clinical status of patients included in our retrospective study, including diuretic therapy. However, CT scans were used as close to the patient’s index diagnosis as possible, and not during an acute admission. Thus, although GGO has less power to discriminate PVOD/PCH, septal thickening and lymphadenopathy remain relevant findings in group 1 PAH patients regardless of the clinical background. Finally, although diagnostic practice patterns differed across the 3 cohorts, with ABG and 6MWT obtained at lower frequency in the Netherlands and Spanish cohorts, the likelihood score was able to maintain performance.

A subset of group 1 patients with significant smoking history (40 pack-years [IQR, 21-50]) and reduced Dl_CO_ (<45%), termed the “lung phenotype,” could confound the PVOD/PCH likelihood score^29^^,^^30^ However, the lung phenotype is clinically distinct from our study population. Our PAH cohort is younger (24.2-54.3 years versus 72 years [IQR, 65-78] for lung phenotype^30^) and predominately female (0-40% male vs 50%-65% for lung phenotype) (Table 2). The lung phenotype can have concurrent radiographic emphysema (49%-62%) or fibrotic changes (30%) and comorbidities like coronary artery disease (27%-42%), all of which we excluded. Lung phenotype patients are often treated less aggressively (82% in COMPERA on single-agent therapy) and less frequently undergo lung transplant. In contrast our patients had severe hemodynamics and required triple therapy, and most required transplant. Critically, lung phenotype patients lack classic WSPH group 1 PAH or PVOD/PCH pathology, exhibiting microvascular medial hypertrophy and not plexiform arteriopathy.^31^ Thus, the PVOD/PCH likelihood score is best applied to younger, transplant-eligible patients with severe hemodynamics and without significant comorbidities.

Several well-known risk factors for pulmonary venous disease are absent from the score: history of CTD-related PAH,^17^^,^^32^ exposures to alkylating chemotherapeutics,^14^^,^^15^^,^^33^ occupational exposures,^13^ hematopoietic stem cell transplantation,^34-36^ and other associated conditions including pulmonary Langerhans cell histiocytosis,^37^ left heart disease,^38^ and sickle cell disease.^39^ The rationale for exclusion is addressed in Table S3. Major themes for exclusion are that (1) a lack of published data in PAH cohorts precludes precise sensitivity or specificity estimation by our methods; (2) our patient registries do not contain full occupational or chemotherapy exposure histories required for validation; and (3) many of these entitles are group 5, not group 1, diagnoses. Since many of the above associated conditions are well-established (eg, alkylating agents), a PVOD/PCH likelihood score should not replace a detailed occupational or drug exposure history. Further, it is established that CTD-PAH patients have high but variable rates of venous disease on pathologic series and therefore should carry a high level of suspicion for PVOD/PCH. We view the PVOD/PCH likelihood score as an adjuvant tool for patient management. Similarly, genetic testing is an important component of PVOD/PCH evaluation to which the score is an adjuvant (Figure 3E). Notably, we include 4 adult patients with the *TBX4* mutation in our cohort who, despite often having complex parenchymal and vascular disease,^40^ have PVOD/PCH likelihood scores between −4 and −8. Finally, we did encounter patients with histologically diagnosed PVOD/PCH who lacked classic features of hypoxemia and radiographic finding. Thus, although rare, false negatives are possible.

It is important that the PVOD/PCH likelihood score is applied to the correct patient population, specifically group 1 PAH patients. The score is not applicable for patients with group 2, 3, 4, or 5 pulmonary hypertension. Presence of ILD in CTD-PAH reduces the predictive ability of the score (Figure S4). For conditions that reduce Dl_CO_, like emphysema or pulmonary edema, we anticipate the score will overcall PVOD/PCH and potentially distract from important management strategies such as left heart–directed therapy. The optimistic discriminatory performance observed for our score in the validation cohorts should be interpreted in the context of deliberate outcome enrichment using histopathology-verified diagnoses. This maximizes signal detection but may not represent routine clinical populations. In this sense, the observed performance reflects the theoretical discriminative ceiling of the selected variables under conditions of minimal outcome misclassification. If the PVOD/PCH likelihood score were applied in non–group 1 PAH patients, or patients with the lung phenotype, anticipated harm could include increased patient anxiety, less aggressive pursuit of vasodilator therapies, or premature referral for lung transplant evaluation. In cases of clinical equipoise, elective inpatient monitoring to assess a patient’s response to vasodilator titrations may help adjudicate a diagnosis and clinical trajectory.

Our PVOD/PCH likelihood score can complement PAH risk assessment tools and provide an additional dimension to assess patients with group 1 PAH (Figure 3D-F). Group 1 patients with a low likelihood of PVOD/PCH fall into group A and are managed as per current PAH guidelines. However, patients who carry higher likelihood of PVOD/PCH stratify into different treatment pipelines, defined by PVOD/PCH likelihood and overall group 1 risk. Intermediate or high PVOD/PCH likelihood may require heightened monitoring upon starting or advancing pulmonary vasodilator therapy. For high-likelihood PVOD/PCH patients, lung transplant evaluation or palliative care counseling is incorporated early. Elective inpatient prostacyclin challenge may be considered as some PVOD/PCH patients tolerate cautious long-term prostacyclin titrations (Figure 3F).

The high discriminatory performance of the score in our validation cohorts does not guarantee clinical usefulness. The score is intended to alert clinicians to a higher likelihood of PVOD/PCH and is not a diagnostic criteria or prescriptive treatment algorithm. Future, longitudinal studies are required to evaluate the effect of the score on transplant referral and evaluation or application for transplant exemption status, justify tailored vasodilator titration, or act as inclusion criteria in clinical trials.

## Supplementary Material

aamag289_Supplementary_Data
