# Supplementary material for: Identification of pulmonary arterial hypertension patients with venous or capillary involvement
Source: Am J Respir Crit Care Med. 2026 Jun 11;212(9):2199–210. doi: 10.1093/ajrccm/aamag289 (PMC13519332; doi:10.1093/ajrccm/aamag289)
Supplement: Supplementary file 1 [file aamag289_supplementary_data.zip › Group1.5LAS_AJRCCM_Online_Supplement.docx]

**Identification of Pulmonary Arterial Hypertension Patients with Venous or Capillary Involvement.**

**Online Supplement.**

**1. Extended Methods:**

**Development of Literature-Derived PVOD/PCH likelihood assessment model**

*Literature review and identification of PVOD/PCH-discriminating variables:*

Studies pertaining to PVOD or PCH were obtained by performing PubMed Medical Subject Headings (MeSH) searches and reviewing citations of returned studies. The following MeSH terms separated by OR statements were used: “Diseases, pulmonary veno occlusive”, “pulmonary veno occlusive disease”, “pulmonary venoocclusive disease”. Search 2 included the MeSH term “pulmonary capillary hemangiomatosis”. We narrowed to “Clinical Trial”, “Meta Analysis,” “RCT,” “Review,” or “Systemic Review” categories. Studies were excluded if they could not be translated to the English language, addressed congenital or acquired pulmonary vein stenosis, or were related to chemotherapy related / transplant related hepatic veno-occlusive disease. We reviewed 164 studies describing veno-occlusive diseases. We further excluded reviews of PVOD or PCH that did not provide patient or population level clinical data, were systematic reviews, or were meta-analyses of patients reported in other studies. For this study, we considered the term ‘Pulmonary Capillary Hemangiomatosis’ clinically synonymous with ‘PVOD’ due to their indistinguishable clinical phenotypes and histopathological overlap^1,2^. We found 76 studies specifically address PVOD or PCH. From these 76 studies, a total of six case control studies^3-8^ reported data from both PVOD/PCH and PAH cohorts. From the 6 case control studies, 32 PVOD/PCH discriminating variables were identified. An additional five case series studies had data specific to PVOD/PCH patients alone and were used in sensitivity and specificity estimates^9-13^.

*Assessment of variable performance:*

Clinical variables were investigated if at least one study presented data comparing PAH to PVOD/PCH for that variable. Once a variable was identified that had at least one study presenting PAH and PVOD/PCH cohort data, further PVOD/PCH cohorts were included to increase overall PVOD/PCH sample size and represent diversity across cohorts for that variable. For example, Monatani et. al. 2008 ^7^ report on BMI for 24 PAH and 24 PVOD patients, making BMI a relevant clinical variable. Luo et. al. 2019 ^9^ also report the BMI of 5 PVOD patients in a case series. Thus, the data from both Montani and Luo are included in the analysis of BMI as a PVOD/PCH discriminating variable. Variables unable to discriminate between PVOD/PCH and PAH in multiple studies (e.g. New York Heart Association Functional Class on Presentation) were excluded from further analysis. In total, 32 variables were identified which had published data for both PAH and PVOD/PCH cohorts. Of these variables, 7 were categorical and 25 were continuous.

*Categorical Variable Assessment:*

For categorical variables, sensitivity and specificity were directly calculated by totaling the number of PVOD/PCH and PAH patients meeting the condition (e.g presence of CT ground glass opacity) across all studies (Supplemental Table 1). After summing this data, sensitivity and specificity were calculated in the standard fashion:

$$Sensitivity= \frac{N PVOD with Condition}{\left( N PVOD with Condition+N PAH with Condition \right)}$$

$$Specificity= \frac{N PAH without Condition}{\left( N PAH without Condition+N PAH without Condition \right)}$$

*Continue Variable Assessment:*

For continuous variables, we generated truncated normal distributions defined by the published mean and standard deviation (or median and range)^14^, pooled these simulated patient distributions for each variable and study, and performed a ROCC analysis^15^ to estimate the Area Under the Curve (AUC) performance, sensitivity, and specificity of a variable as a function of cut-point. The ROCC cut-point maximizing sensitivity and specificity, known as Youden’s Point (YP) was selected^16^. Variables with high statistical performance (ROCC AUC > 0.9 and sensitivity and specificity > 85%) such as DLCO percent predicted or PaO2 on ABG allowed the use of two cut-points– one maximizing sensitivity for PVOD/PCH and another maximizing sensitivity for PAH. This process is illustrated in **Supplemental Figure 1** for percent predicted DLCO and ABG PaO2 is shown in detail for each variable in each study in **Supplemental Figure 2**.

In studies, continuous variables data is presented predominately as mean and standard deviation of distributions except for Ogawa et. al. 2018 ^6^ and Holcomb et al. 2000 ^12^. In Ogawa, some data were reported as a median and range (e.g. median six-minute walk test distance was 128 m with a range of 0–360 meters). For these variables, the methodology outlined by Hozo ^17^ was used to estimate the mean and standard deviation from the median, range, and sample size. In Holcomb et al, the mean and variance were calculated directly from discrete data. Because mean and standard deviation are published in the literature, a gaussian data distribution to the data is assumed. However, some variable data have skew. For instance, pack years of smoking is presented as 2.7 ± 5.1 Pack Year for PAH and 13.6 ± 16.9 for PVOD ^7^; skew is present as negative pack years are not possible.

To circumvent this, we simulated distributions using a truncated normal distribution with the “truncnorm” package in R^18^. Briefly, a standard random normal distribution is generated using the specified mean and standard deviation. However, an upper or lower boundary for the distribution is specified (e.g. 0 pack-years). An acceptance and rejection algorithm includes the randomly generated data value if it falls within the specified boundaries^14^. Resulting distributions are normal, are constrained to relevant clinical boundaries, match published mean and standard deviation values, and contain a desired number of subjects. This method was chosen because skew could not be inferred from the available data. For most variables (e.g. Age, BMI) the lower bound for the truncated normal distribution was chosen to be 0, and the upper boundary was not limited. For variables dealing with percentage (e.g. SpO2) the boundaries of the truncated normal distribution were chosen to be 0 and 100. Finally, since DLCO can be greater than 100% of predicted, only a lower bound of 0% was used for the truncated normal distribution.

We investigated three methods to set the number of patients in each simulated distribution, ultimately settling on uniformly multiplying the number of patients in each study by 100 to both ensure a smooth ROCC analysis and ensure that each study was weighted appropriately in the final summed patient distribution.

For the first method, the number of simulated patients exactly matched the number of published patients. In this case, because Montani et. al. 2008 reported on 24 PVOD or PAH patients, the simulated distribution for pack years of smoking for PVOD patient would consist of 24 values bounded by 0 with a mean of 13.6 and standard deviation of 16.9 pack years. However, since the resolution of the ROC and CDA curves is determined by the number of patients in the analysis, low patient numbers generate variability in downstream analysis due to random sampling. We verified this by running simulations varying the number of patients over a range starting from 1 to 2500. As shown in **Supplemental Figure 10**, the ROC AUC and Youden’s point converge to a final value as patient values increase and the functions become smoother. Thus, the results produced by the ROC analysis methodology (AUC, Sensitivity and Specificity curves) are a function of only mean and standard deviation. Error caused by random sampling is minimized provided the number of simulated patients is sufficiently large. Increasing the number of simulated patients causes the simulated distribution to converge on a smooth function for the Gaussian patient distribution, ROC, and CDA curves (**Supplemental Figure 1**).

For the second method, we generated distributions containing 1000 patients per study. However, this overrepresented studies with fewer patient numbers in the summed final distributions.

For the third method used on our analysis, we multiplied the number of patients reported in each study by 100 to determine the number of patients in each distribution. In the example of Montani et. al 2008, since 24 PVOD patients are reported on in the study, 2400 simulated patients were used to make the simulated PVOD/PCH distribution. In this manner we maintain the proportional contribution of larger and smaller studies in the final pooled distribution. ROC and CDA analyses were performed for each variable for individual study and across study distributions in R (**Supplemental Figure 2**). Cut-points (the test value used to discriminate between PVOD/PCH and PAH) were determined by Youden’s point (the point on the ROC curve corresponding which maximizes Sensitivity + Specificity-1 ^16^) for variables with ROCC AUC < 0.9 and sensitivity or specificity < 85%. The performance of DLCO percent predicted and ABG PaO2 exceeded these thresholds, and allowed us to choose two cut points, one to maximize sensitivity and one to maximize specificity (**Supplemental Figure 1**). All above calculations were conducted in R (2023, Vienna Austria).

*Derivation of likelihood scoring framework:*

PVOD/PCH discriminators having an estimated sensitivity or specificity >70% were selected for inclusion in the likelihood score. We elected to use a point system that weighted variables by their statistical performance. Variables with an estimated sensitivity or specificity ≥80% are weighted by two points, whereas variables with a sensitivity or specificity of 70-79% are weighted by one point. For continuous variables, positive points were given for patient variable values meeting the ROCC derived sensitivity cut point for PVOD/PCH. Negative points were assigned for patient variable values meeting the specificity cut point for PVOD/PCH. Categorical variables were assigned either positive or negative points for the presence or absence of the condition. This scoring framework was employed to cumulatively assign points to each patient based on criteria met across the set of discriminating variables. A positive total score was designed to signal greater likelihood of PVOD/PCH. In derivation of this likelihood prediction score, we chose to omit variables that were clinically invasive or had performance informed by only one study (e.g. hemosiderin laden macrophages on bronchoalveolar lavage or mean systemic arterial pressure on right heart catheterization). We excluded DLCO adjusted for alveolar volume (DLCO/VA) as percent predicted DLCO had better performance and was supported by more studies. Finally, we omitted age from the scoring model due to studies reporting a bimodal age distribution in PVOD/PCH^8^. Finally, associated conditions (e.g., connective tissue disease) and exposures (e.g., chemotherapy agents, organic solvents) were recognized by studies in our literature review as predisposing factors for PVOD/PCH. Although these factors can help physicians assess clinical likelihood of PVOD/PCH, they were excluded from our score derivation either because (1) quantifying their discriminatory performance was precluded due to a lack of a published comparator group in the existing literature (inability to perform sensitivity/specificity calculations) or (2) data were not documented among cohorts utilized to test the likelihood score. This is detailed in **Supplemental Table 3**.

*Sensitivity Analysis:*

To assess whether the heuristic weighting scheme outlined above influenced score performance, we conducted additional sensitivity analyses using three alternative score derivation strategies, that varied both inclusion thresholds and weighting approaches for variables. Each method and it’s overall performance is tabulated in **Supplemental Table 6**. Method A is the method described above and used in the paper that weighs variables with a Sensitivity (Sn) or Specificity (Sp) > 80% with +/- 2 points. Variable with a Sn or Sp between 70-80% are weighted with +/- 1 point. For Method B, we weighted +/- 1 point for all variables with an Sn or Sp > 70%. For Method C, we only used the highest performing variables (Sn or Sp > 80%) and weighted with +/- 1 point. Finally, for Method D, we used variables with Sn and Sp > 70% and weighted each variable by their average Sn or Sp. In this case the weighting would be 0.905 for DLCO, or 0.89 for lowest SpO2 for six-minute walk test. The R pROC package^19^ was used to assess the ROCC AUC performance of each method at discriminating cases (PVOD/PCH) from controls (IPAH^+^ and HPAH) in all three combined cohorts. The pROC package was also used to calculate a 95% bootstrap confidence interval around the AUC, and 95% confidence interval bounds on sensitivity and specificity.

*Limitations to the Sensitivity and Specificity Estimates:*

It is possible that the same patients may be included in multiple PVOD/PCH studies, leading to their double-counting in our sensitivity and specificity estimates. This is a limitation of not having access to granular, patient level data in published PVOD/PCH studies. We considered trying to exclude chronologically overlapping studies, but there was no way to feasibly decide which studies to include and which to reject. Further, this would likely exclude novel patients that were included in one study and not another. As such, our sensitivity and specificity values are the best estimates that we can provide given the data available.

**Multi-center evaluation of PVOD/PCH likelihood score performance**

To evaluate the PVOD/PCH discrimination accuracy of the literature-derived likelihood score, we assembled (i) an internal test cohort from our Stanford PH observational database and (ii) independent external validation cohort from two different observational registries in Europe. All PVOD/PCH and non-PVOD/PCH PAH cases were confirmed by histopathology. All subjects had hemodynamics on RHC consistent with WHO Group 1 PAH based on guidelines at the time of diagnosis. We excluded subjects with pre-existing conditions generating peripheral cyanosis (e.g., large intracardiac shunts, atrial septostomy, or Eisenmenger’s physiology), multifactorial mixed-phenotype PH (e.g., Group 1 overlap with Group III or IV PH), or excessive missing data. STROBE diagrams for each cohort are shown in **Supplemental Figure 3**.

*Stanford Validation Cohort:*

Between 1995 and 2023, Group 1 PAH cases at Stanford University Hospital were retrospectively reviewed for histologically confirmed PVOD/PCH and idiopathic PAH (termed “IPAH^+^”) cases. Additional cases without verified pathology were included in the Stanford cohort. These patients included heritable PAH patients with either a familial inheritance pattern or *BMPR2^+/-^* mutation status (HPAH), idiopathic PAH patients stable on high dose, long-term prostacyclin therapy (IPAH), PAH associated with mixed connective tissue disease without (APAH-CTD) and with radiographic evidence of fibrotic ILD (APAH-CTD-ILD). For all primary cohorts, we excluded patients with either significant restriction and radiographic fibrosis or significant obstructive lung disease (defined as FEV1/FVC < 0.70 and FEV1 < 0.60). For all patients, clinical variables (PFT, CT, 6-minute walk test) were obtained as close to the time of diagnosis (defined as index right heart catheterization) as possible.

To determine the presence or absence of specific radiographic features in the Stanford PVOD/PCH, and IPAH^+^ cohorts, CT scans were reviewed using PACS DICOM viewer Sectra (Linköping, Sweden) by two independent radiologists (H.H.G. 14 years’ experience; and I.Y.Y. 5 years’ experience) who were blinded to the underlying diagnosis. Thin (1-1.25mm) axial cuts were viewed using mediastinal soft tissue (400 width, 40 level) and lung (1500 width, -700 level) windows. The presence or absence of imaging features were assessed independently and agreed upon by consensus. Specific radiographic features were also determined. The presence of fibrotic ILD was determined by observing honeycombing, traction bronchiectasis, traction bronchiectasis, architectural distortion, or reticulation in non-dependent areas. Mediastinal lymphadenopathy was determined if there were lymph nodes with a with short axis greater than 10mm in diameter ^20^. For the remaining Stanford patient groups (IPAH, HPAH, APAH-CTD, and APAH-CTD-ILD), CT scan reports were parsed for direct mention of any form of “centrilobular ground glass opacity”, “lymphadenopathy” and “septal line thickening.” The presence of fibrotic interstitial lung disease was also assessed in all patient CT scans.

*Madrid, Spain and Amsterdam, Netherlands Cohorts:*

The external validation cohort was made up of cases from (1) the Hospital Universitario 12 de Octubre, Madrid, Spain from 2010 to 2022 and (2) the Amsterdam UMC Center for PH Excellence, Netherlands from 1996 to 2023. Here, all PVOD/PCH and idiopathic PAH cases have verified histopathology (again termed IPAH^+^). HPAH cases either have validated histopathology with an established family inheritance pattern or a known *BMPR2* (n = 25) or *TBX4* (n = 4) heterozygous mutation. All cases in the validation cohort use the radiologist’s CT report to determine the presence or absence of CT features. All data included in the trial were obtained as close to the index diagnosis of PAH by right heart catheterization as possible, prior to therapy initiation. *A priori* criteria were set for patient exclusion due to excessive missing data. Patients without a CT scan were only included in the study if ABG, 6MWT, DLCO, sex, and smoking history data were all available (N = 2). Among subjects with complete chest CT data, we included those with availability of at least two of three oxygenation parameters (ABG, 6 MWT or DLCO) and one of two patient level parameters (Sex or Pack Years Smoking). See **Supplemental Table 4**. Similar to the Stanford USA cohort, patients with significant obstruction or fibrotic lung disease were excluded.

For all cohorts, the study was approved by each Institutions Internal Review Board (Stanford, Hospital Universitario 12 de Octubre, Madrid, Spain) or the Medical Ethics Review Committee (Amsterdam VU University Medical Center 2018-613) with tissue collection performed under informed consent.

*Missing Data Imputation:*

Missing data was handled by zero imputation due to its feasibility for clinical practice. Multivariate predictive mean matching imputation was also implemented via the R MICE^21^ package, and the resultant PVOD/PCH Likelihood scores were compared to zero imputation.

*Assessment of PVOD/PCH score discrimination accuracy:*

The PVOD/PCH Likelihood Score was applied to all three cohorts. For the Stanford test and Netherlands validation cohorts, a 1-Way ANOVA was used to compare the effect of patient classification on the PVOD/PCH Likelihood Score. P values were generated with a post-hoc Tukey test. For the Spain Validation Cohort, P values are calculated using a Welch Two Sample T-test. To determine overall score performance, IPAH^+^ and HPAH patients from all three cohorts were pooled into a single “PAH” cohort that was compared to all PVOD/PCH patients from the three cohorts. Comparing PAH and PVOD/PCH, the mean ±95% confidence interval (CI) AUC performance of the score was determined by ROCC analysis with bootstrapping in the method of DeLong^22^ using the R package pROC^23^. Bootstrapping ROCC analysis was also used to assess the mean ±95% CI AUC performance of each PVOD/PCH Likelihood score component compared to the full PVOD/PCH Likelihood Score and assess the effect of removing each score component from the PVOD/PCH Likelihood Score. Sensitivity and specificity ±95% confidence interval for PVOD/PCH in the pooled study population was calculated at each PVOD/PCH Likelihood Score value as was the score positive predictive value (PPV) and negative predictive value (NPV). Because PPV and NPV depend on prevalence, we calculated the NPV and PPV for PVOD/PCH prevalence rates of 1%, 5%, and 10% given the estimated PVOD/PCH prevalence of 5-10% in PAH ^24^ using the following equations:

$$PPV= \frac{\left( SN\times P \right)}{\left\{ \left( SN\times P \right)+\left[ \left( 1-SP \right)\times\left( 1-P \right) \right] \right\}}$$

$$NPV= \frac{SP\left( 1-P \right)}{\left\{ SP\left( 1-P \right)+P\left( 1-SN \right) \right\}}$$

Where SN = sensitivity, SP = specificity, and P = prevalence. Because prevalence for PVOD/PCH in PAH is estimated to be between 5% and 10%, we used 1%, 5%, and 10% for prevalence in our calculations. Statistical analyses were conducted in R (2023, Vienna Austria).

*Calibration Analysis:*

To assess agreement between predicted and observed PVOD/PCH probabilities, we fitted a generalized linear model relating dichotomous PVOD/PCH status (0 or 1 outcome) to the PVOD likelihood score, using the ‘glm’ function within the ‘stats’ R software package (version 3.6.2). The final model was of the form p = 0.558 + (0.0656 * Score). Next the function “hoslem.test” from the package ‘ResourceSelection’ (version 0.3-6) was applied to calculate the Hosmer-Lemeshow statistic and p-value. Note, the Hosmer-Lemeshow test quantified goodness-of-fit by splitting the data into groups with differing predicted probabilities across the range of probabilities in the population (e.g., first group with lowest probability, second group with next lowest probability, and so on, split into probability deciles). Within each group, the test compared the predicted probability and observed proportion of PVOD/PCH, arriving at a statistic with a chi-square distribution. Practically speaking, the Hosmer-Lemeshow test can be thought of as a lack-of-fit test, whereby the null hypothesis (large p-value) indicates a good fit (the observed PVOD/PCH rates match predicted probabilities across groups) and the alternative hypothesis (small p-value) indicates poor fit (the observed PVOD/PCH rates differ from probabilities more than would be expected by chance). To visualize the Hosmer-Lemeshow results, we implemented the function ‘calibration.plot’ from the github repository ‘RPMH_Resources’ R package, the results of which are presented in **Supplemental Figure 7**, plotting observed PVOD/PCH proportions versus predicted probabilities binned by predicted probability groups. A simplified calibration bar plot was created in R to show the proportion of observed PVOD/PCH cases as a function of PVOD/PCH Likelihood score, **Figure 3A.**

**Integration of PVOD/PCH Likelihood into Existing PAH Management:**

Contemporary evidence suggests that slow and cautious vasodilator therapy may be tolerated in PVOD/PCH patients ^9,25,26^. The clinical pipelines suggested in Figure 2E-F were developed thorough expert consensus (R.L., Y.K.L., K.K., Y.S., E.S., V.D.J.P., and R.Z.) at Stanford University.

**2. Online Supplement Figure Captions:**

**Supplemental Figure 1:** Example of literature-derived simulated distributions and Receiver Operator Characteristics Curve (ROCC) analysis for percent predicted Diffusion capacity of the Lung for Carbon monoxide (DLCO). **A.** Reported statistical data from eight studies are used to simulate truncated normal distributions of DLCO. The number of patients in each distribution is proportional to the number reported in the study. A sufficiently high number of patients is chosen to ensure a smooth ROCC curve with sensitivity and specificity estimation convergence (see **Supplemental Figure 10**). The red line indicates Youden’s Point (YP) and blue lines indicate cut points chosen to maximize sensitivity and specificity. **B.** ROCC analysis of the total summed Pulmonary Veno-Occlusive Disease (PVOD) and Pulmonary Arterial Hypertension (PAH) distributions in **A**. The DLCO cut point of 52% corresponds with Youden’s Point, the point of maximum sensitivity and specificity. **C.** Cumulative Data Analysis curve from the ROCC analysis in **B** shows sensitivity and specificity for PVOD as a function of the DLCO cut point. Note that by convention, sensitivity and specificity are calculated from the subset of the distribution to the left of the cut point. The red line indicates Youden’s Point (YP), blue lines indicate cut points chosen to maximize sensitivity and specificity. A detailed report of simulated distributions, ROCC Area Under the Curve (AUC) sensitivity, and specificity on a per study basis for each variable is given in **Supplemental Figure 2**. **D:** Similar analysis for Arterial Blood Gas (ABG) Partial Pressure of Oxygen in Arterial Blood (PaO2). Here six total studies contribute to the simulated distributions in **D** and the ROCC AUC (area under the curve) is equal to 0.84, **E.** Youden’s point corresponds to 67 mmHg in **F.** For the purposes of our study, cut points of 60 and 70 mmHg were chosen to increase sensitivity and specificity to 0.85 and 0.90, respectively

**Supplemental Figure 2:** Summary of all continuous variable sensitivity and specificity estimations for each variable and each study. The left columns (Simulated Distributions) show the simulated distributions as calculated from the study reported mean and standard deviations for both PAH and PVOD/PCH patients. The line graphs define the extent of the mean ± standard deviation. The calculated Youden’s Point is shown as a black dot. The middle columns (ROCC AUC) report the area under the curve for each ROCC analysis performed on the study distributions. Open diamonds indicate the ROCC AUC for an individual study. Closed diamond indicates the summary ROC AUC calculated from the sum of all patient populations (denoted as “Summary”). The right sided columns (Sensitivity and Specificity at YP) plot the Sensitivity and Specificity at Youden’s point as a bar graph for each study individually and in aggregate (“Summary”). The black numbers the left of the bar graphs reports the value of the cut point corresponding to Youden’s point. YP = Youden’s Point.

**Supplemental Figure 3:** STROBE Flow Charts for the Stanford, Spain, and Netherlands Patient Cohorts.

**Supplemental Figure 4:** Comparison PVOD/PCH likelihood scores between primary and secondary cohorts in data from the Stanford, USA center. PVOD/PCH indicates pathologically validated PVOD/PCH patients undergoing blinded radiologist review of CT scans or independent review of original CT report. IPAH^+^ indicates pathologically validated IPAH patients undergoing blinded radiologist review of CT scans or independent review of original CT report. HPAH indicates heritable PAH patients with established mutation (*BMPR2* or *TBX4*) or an established familial PAH history. IPAH indicates PAH patients on long term prostacyclin therapy but without pathologic verification. APAH-CTD indicates PAH patients associated with connective tissue disease but without pathologic verification and without radiographic evidence of fibrotic lung disease on chest CT. APAH-CTD-ILD indicates PAH associated with connective tissue disease, evidence of fibrotic interstitial lung disease on CT, and no pathologic verification. Blue boxplots indicate cases (PVOD/PCH) used in subsequent analysis. White boxes indicate controls (IPAH^+^ or PVOD/PCH) used in subsequent analysis. Grey boxes indicate secondary cohorts (no pathologic validation) which are not used in any subsequent receiver operator characteristics curve or calibration analyses. Red dots indicate CT scan assessment by two radiologists blinded to the diagnosis and in agreement of the findings. Grey dots indicate CT findings obtained from the CT report. Finally, black dots indicate secondary cohort members where CT findings were obtained from the CT report.

**Supplemental Figure 5: A.** The PVOD/PCH Likelihood score maintains its performance when data is missing or omitted from the score (95% bootstrap AUC CI shown), again comparing combined IPAH^+^ and HPAH classes to PVOD/PCH. **B.** The PVOD/PCH Likelihood score outperforms any single component of the likelihood score.

**Supplemental Figure 6:** Overview of missing data in the three cohorts and overview of score performance with different imputation methods for missing data. **A.** Heatmap showing the distribution of missing data for all patients included in the study. **B.** Comparison of imputation methods for missing data. Clinically relevant zero imputation of missing data (orange boxes) is compared to predictive mean matching imputation of missing data (green boxes). Overall, there are minor changes in the score distribution between the two imputation methods.

**Supplemental Figure 7:** Calibration plot and Homser-Lemeshow analysis of the PVOD/PCH Likelihood Score. A generalized linear model relating dichotomous PVOD/PCH status (0 or 1 outcome) to PVOD likelihood score was created. The final model was of the form p = 0.558 + (0.0656 * Score). Next the Hosmer-Lemeshow statistic and p-value were calculated, where data are split into deciles with rising probability of PVOD/PCH. The null hypothesis is that there is no difference, and therefore a good fit, between observed PVOD/PCH rates in each decile and predicted PVOD/PCH probability. The alternative hypothesis is that observed PVDO/PCH rates in each decile deviate from predicted more than expected by chance. The Hosmer-Lemeshow goodness of fit χ^2^ statistic was 5.78, with a p-value of 0.672, indicating a good fit between the risk prediction score and the binary outcome of PVOD/PCH versus PAH.

**Supplemental Figure 8:** Sensitivity analysis of different PVOD/PCH Likelihood Score Methods. Details for each score method as well as performance and score range are tabulated in **Supplemental Table 6.** Each score method is analyzed by Receiver Operator Characteristic Curve analysis comparing PVOD/PCH as cases to PAH controls (IPAH^+^ and HPAH combined cohorts). Area Under the Curve (AUC) +/- 95% bootstrap confidence interval is shown. Blue lines and shaded regions represent 95% confidence interval bounds around sensitivity (dashed lines) and specificity (dotted lines).

**Supplemental Figure 9:** Percent allocation of all patients (PAH vs. PVOD/PCH) in the three study cohorts as a function of the low and high cut-points. **A.** The low cut-point separates low likelihood PVOD/PCH patients from intermediate likelihood PVOD/PCH patients. **B.** The high cut-point separates intermediate likelihood PVOD/PCH patients from high likelihood PVOD/PCH patients. By convention, the low cut-point categorizes all patients with Likelihood scores less than or equal to the cut point as low-likelihood PVOD/PCH. Similarly, the high cut-point categorizes all patients with a Likelihood score greater than or equal to the cut point as high-likelihood PVOD/PCH. This is why the graphs in A and B are similar but shifted by one score point.

**Supplemental Figure 10:** The results of the ROCC analysis (Supplemental Figure 1 and 2) on simulated truncated normal distributions is a function of the mean and standard deviation of the distribution if the number of patients in each distribution is sufficiently high. **A.** Example of simulated truncated normal distributions of percent predicted DLCO values for PAH (orange) and PVOD/PCH (green) patients using the mean (70.5% for PAH, 51.9% for PVOD/PCH) and standard deviation (15.2% for PAH, for PVOD/PCH 19.3%) reported in Montani 2008 ^7^. The distribution converges to a smooth Gaussian distribution with higher simulation numbers. **B.** ROCC AUC and Youden’s Point **C** as a function of the number of patients used in the simulation. As shown, both ROCC AUC and Youden’s point converge to values of 0.77 and 61% respectively with patient populations over 1000. Red lines indicate a running average of 10 values. Blue lines indicate a running average ± one standard deviation of 10 values.

**3. Extended Method References:**

1.	Chaisson NF, Dodson MW, Elliott CG. Pulmonary Capillary Hemangiomatosis and Pulmonary Veno-occlusive Disease. *Clin Chest Med* 2016; **37**(3): 523-34.

2.	Weatherald J, Dorfmuller P, Perros F, et al. Pulmonary capillary haemangiomatosis: a distinct entity? *Eur Respir Rev* 2020; **29**(156).

3.	Hadinnapola C, Bleda M, Haimel M, et al. Phenotypic Characterization of EIF2AK4 Mutation Carriers in a Large Cohort of Patients Diagnosed Clinically With Pulmonary Arterial Hypertension. *Circulation* 2017; **136**(21): 2022-33.

4.	Seferian A, Helal B, Jais X, et al. Ventilation/perfusion lung scan in pulmonary veno-occlusive disease. *Eur Respir J* 2012; **40**(1): 75-83.

5.	Rabiller A, Jais X, Hamid A, et al. Occult alveolar haemorrhage in pulmonary veno-occlusive disease. *Eur Respir J* 2006; **27**(1): 108-13.

6.	Ogawa A, Takahashi Y, Matsubara H. Clinical prediction score for identifying patients with pulmonary veno-occlusive disease/pulmonary capillary hemangiomatosis. *J Cardiol* 2018; **72**(3): 255-60.

7.	Montani D, Achouh L, Dorfmuller P, et al. Pulmonary veno-occlusive disease: clinical, functional, radiologic, and hemodynamic characteristics and outcome of 24 cases confirmed by histology. *Medicine (Baltimore)* 2008; **87**(4): 220-33.

8.	Montani D, Lau EM, Descatha A, et al. Occupational exposure to organic solvents: a risk factor for pulmonary veno-occlusive disease. *Eur Respir J* 2015; **46**(6): 1721-31.

9.	Luo Q, Jin Q, Zhao Z, et al. Targeted therapy in pulmonary veno-occlusive disease: time for a rethink? *BMC Pulm Med* 2019; **19**(1): 257.

10.	Montani D, Girerd B, Jais X, et al. Clinical phenotypes and outcomes of heritable and sporadic pulmonary veno-occlusive disease: a population-based study. *Lancet Respir Med* 2017; **5**(2): 125-34.

11.	Lantuejoul S, Sheppard MN, Corrin B, Burke MM, Nicholson AG. Pulmonary veno-occlusive disease and pulmonary capillary hemangiomatosis: a clinicopathologic study of 35 cases. *Am J Surg Pathol* 2006; **30**(7): 850-7.

12.	Barry W. Holcomb J, MD; James E. Loyd, MD; E. Wesley Ely, MD, FCCP; Joyce Johnson, MD; and Ivan M. Robbins, MD. Pulmonary Veno-occlusive Disease A Case Series and New Observations. *Chest* 2000; **118**: 1671-9.

13.	Boucly A, Solinas S, Beurnier A, et al. Outcomes and risk assessment in pulmonary veno-occlusive disease. *ERJ Open Res* 2024; **10**(1).

14.	Geweke J. Efficient simulation from the multivariate normal and Student-t distributions subject to linear constraints and the evaluation of constraint probabilities. In: Keramidas EM, ed. Computing Science and Statistics: Proceedings of the 23rd Symposium on the Interface. Fairfax Station, VA: Interface Foundation of North America, Inc.; 1991: 571–8.

15.	Peterson W, Birdsall T, Fox W. The theory of signal detectability. *Transactions of the IRE Professional Group on Information Theory* 1954; **4**(4): 171-212.

16.	Youden WJ. Index for rating diagnostic tests. *Cancer* 1950; **3**(1): 32-5.

17.	Hozo SP, Djulbegovic B, Hozo I. Estimating the mean and variance from the median, range, and the size of a sample. *BMC Med Res Methodol* 2005; **5**: 13.

18.	Mersmann O TH, Steuer D, Bornkamp B. _truncnorm: Truncated Normal Distribution_. . 2023. <https://CRAN.R-project.org/package=truncnorm>.

19.	Robin X, Turck N, Hainard A, et al. pROC: an open-source package for R and S+ to analyze and compare ROC curves. *BMC Bioinformatics* 2011; **12**: 77.

20.	Sharma A, Fidias P, Hayman LA, Loomis SL, Taber KH, Aquino SL. Patterns of lymphadenopathy in thoracic malignancies. *Radiographics* 2004; **24**(2): 419-34.

21.	Buuren Sv, Groothuis-Oudshoorn K. mice: Multivariate Imputation by Chained Equations inR. *Journal of Statistical Software* 2011; **45**(3).

22.	DeLong ER, DeLong DM, Clarke-Pearson DL. Comparing the areas under two or more correlated receiver operating characteristic curves: a nonparametric approach. *Biometrics* 1988; **44**(3): 837-45.

23.	Nossent EJ, Smits JA, Seegers C, et al. Clinical Correlates of a Nonplexiform Vasculopathy in Patients With a Diagnosis of Idiopathic Pulmonary Arterial Hypertension. *Chest* 2024; **166**(1): 190-200.

24.	Montani D, Price LC, Dorfmuller P, et al. Pulmonary veno-occlusive disease. *Eur Respir J* 2009; **33**(1): 189-200.

25.	Ogawa A, Sakao S, Tanabe N, Matsubara H, Tatsumi K. Use of vasodilators for the treatment of pulmonary veno-occlusive disease and pulmonary capillary hemangiomatosis: A systematic review. *Respir Investig* 2019; **57**(2): 183-90.

26.	Humbert M, Kovacs G, Hoeper MM, et al. 2022 ESC/ERS Guidelines for the diagnosis and treatment of pulmonary hypertension. *Eur Respir J* 2023; **61**(1).
