# Supplementary material for: Identification of pulmonary arterial hypertension patients with venous or capillary involvement
Source: Am J Respir Crit Care Med. 2026 Jun 11;212(9):2199–210. doi: 10.1093/ajrccm/aamag289 (PMC13519332; doi:10.1093/ajrccm/aamag289)
Supplement: Supplementary file 1 [file aamag289_supplementary_data.zip › Group1.5LAS_Supplemental_Figures.pdf]

Supplemental Table 1: Categorical Variable Sensitivity and Specificity Estimation

			Reported		Totals		Test/Variable Positive		Test/Variable Negative			
			Reported	Total	PAH	PVOD/ PCH	PAH	PVOD/ PCH	PAH	PVOD/ PCH	Sensitivity	Specificity
**Variable**	**Study**	**Source**	**P Value**	**Studies**	*A*	*B*	*C*	*D*	*E*	*F*	*D/B*	*E/A*
**Male Sex**	Montani, 2015	France	0.0001		65	33	20	27	45	6	0.82	0.69
	Montani, 2008	France	0.02		24	24	4	12	20	12	0.50	0.83
	Ogawa, 2018	Japan	0.001		55	19	15	14	40	5	0.74	0.73
	Lantuejoul, 2006	UK			0	35	0	22	0	13	0.63	
	Rabiller, 2006	France	1		11	8	3	5	8	3	0.63	0.73
	Luo, 2019	China			0	5	0	4	0	1	0.80	
	Holcomb, 2000	USA			0	11	0	5	0	6	0.45	
	Seferian, 2012	France	0.003		70	56	27	37	43	19	0.66	0.61
	Hadinnapola, 2017	UK	0.001		704	16	210	7	494	9	0.44	0.70
	Montani, 2017 - *EIF2AK4* Mutants	France			0	27	0	13	0	14	0.48	
	Montani, 2017 - WT	France			0	67	0	48	0	19	0.72	
	Boucly, 2024	France			0	327	0	227	0	100	0.69	
	**Aggregate Total**			**12**	**929**	**628**	**279**	**421**	**650**	**207**	**0.67**	**0.70**
**Never Smoker**	Montani, 2015	France	0.0001		65	33	39	5	26	28	0.15	0.40
	Montani, 2008	France	0.15		21	21	14	9	7	12	0.43	0.33
	Ogawa, 2018	Japan	0.001		55	19	46	6	9	13	0.32	0.16
	Rabiller, 2006	France			11	8	7	2	4	6	0.25	0.36
	Luo, 2019	China			0	5	0	3	0	2	0.60	
	**Aggregate Total**			**5**	**152**	**86**	**106**	**25**	**46**	**61**	**0.29**	**0.30**
**Lymphadenopathy**	Montani, 2008	France	0.005		13	20	1	13	12	7	0.65	0.92
**Chest CT**	Ogawa, 2018	Japan	0.02		55	19	4	6	51	13	0.32	0.93
	Luo, 2019	China			0	5	0	1	0	4	0.20	
	Resten, 2004	France	0.0001		15	15	0	12	15	3	0.80	1.00
	Holcomb, 2000	USA			0	9	0	3	0	6	0.33	
	Hadinnapola, 2017	UK	0.001		21	14	0	11	21	3	0.79	1.00
	**Aggregate Total**			**6**	**104**	**82**	**5**	**46**	**99**	**36**	**0.56**	**0.95**
**Centrilobular GGO**	Montani, 2008	France	0.05		13	20	3	13	10	7	0.65	0.77
**Chest CT**	Ogawa, 2018	Japan	0.001		55	19	21	19	34	0	1.00	0.62
	Luo, 2019	China			0	5	0	5	0	0	1.00	
	Resten, 2004	France	0.003		15	15	5	13	10	2	0.87	0.67
	Holcomb, 2000	USA			0	9	0	5	0	4	0.56	
	Hadinnapola, 2017	UK			21	14	3	4	18	10	0.29	0.86
	**Aggregate Total**			**6**	**104**	**82**	**32**	**59**	**72**	**23**	**0.72**	**0.69**
**Septal Line Thickening**	Montani, 2008	France	0.01		13	20	2	13	11	7	0.65	0.85
**Chest CT**	Ogawa, 2018	Japan	0.001		55	19	8	17	47	2	0.89	0.85
	Luo, 2019	China			0	5	0	4	0	1	0.80	
	Resten, 2004	France	0.0001		15	15	2	14	13	1	0.93	0.87
	Holcomb, 2000	USA			0	9	0	6	0	3	0.67	
	Hadinnapola, 2017	UK			21	14	1	9	20	5	0.64	0.95
	**Aggregate Total**			**6**	**104**	**82**	**13**	**63**	**91**	**19**	**0.77**	**0.88**
**Pleural Effusion**	Montani, 2008	France			13	20	2	4	11	16	0.20	0.85
**Chest CT**	Ogawa, 2018	Japan	0.168		55	19	4	4	51	15	0.21	0.93
	Resten, 2004	France	0.65		15	15	2	4	13	11	0.27	0.87
	**Aggregate Total**			**3**	**83**	**54**	**8**	**12**	**75**	**42**	**0.22**	**0.90**
**Pericardial Effusion**	Montani, 2008	France	0.05		13	20	6	2	7	18	0.10	0.54
**Chest CT**	Resten, 2004	France			15	15	9	9	6	6	0.60	0.40
	Holcomb, 2000	USA			0	9	0	2	0	7	0.22	
	**Aggregate Total**			**3**	**28**	**44**	**15**	**13**	**13**	**31**	**0.30**	**0.46**

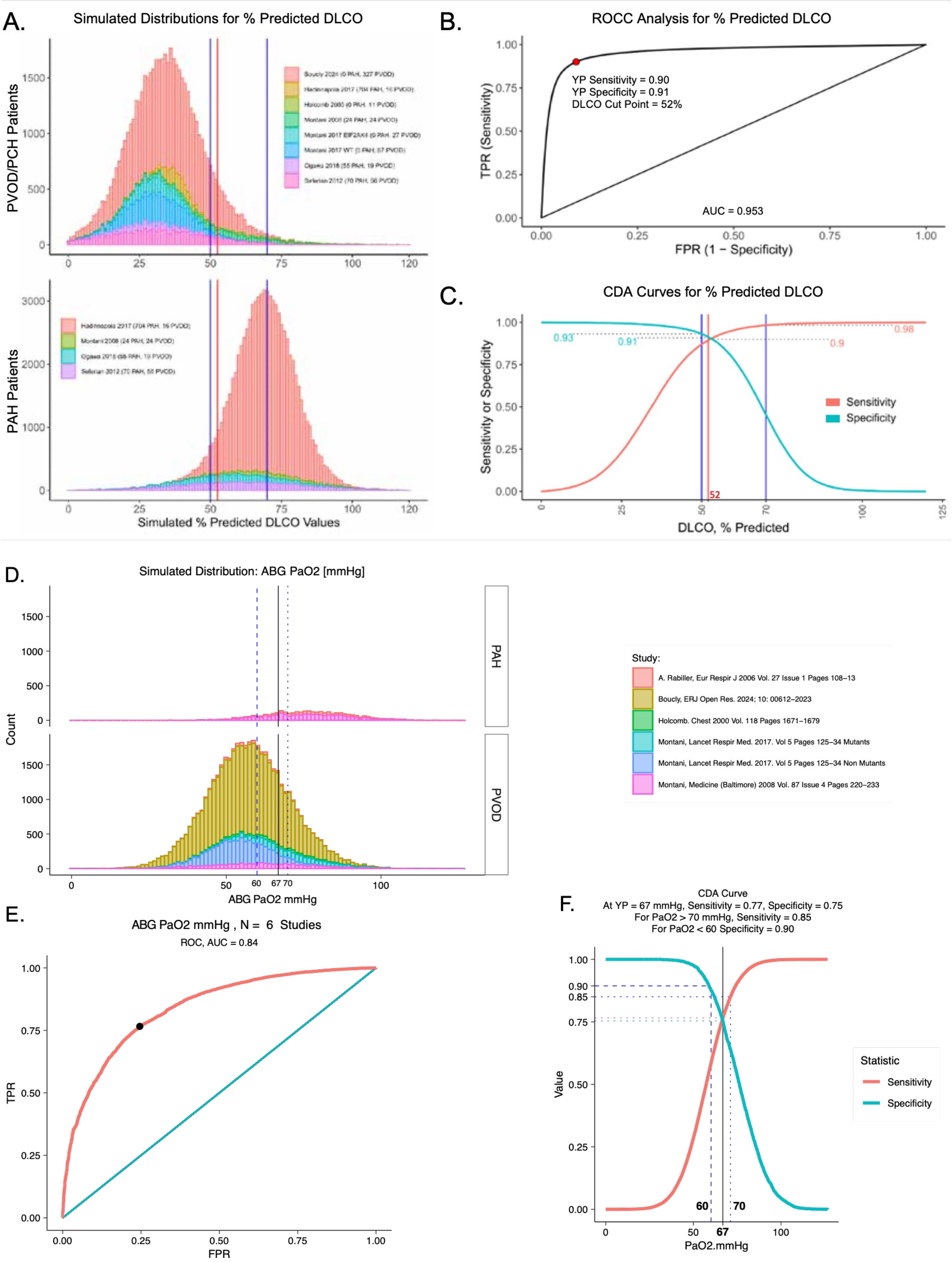

Supplemental Figure 1

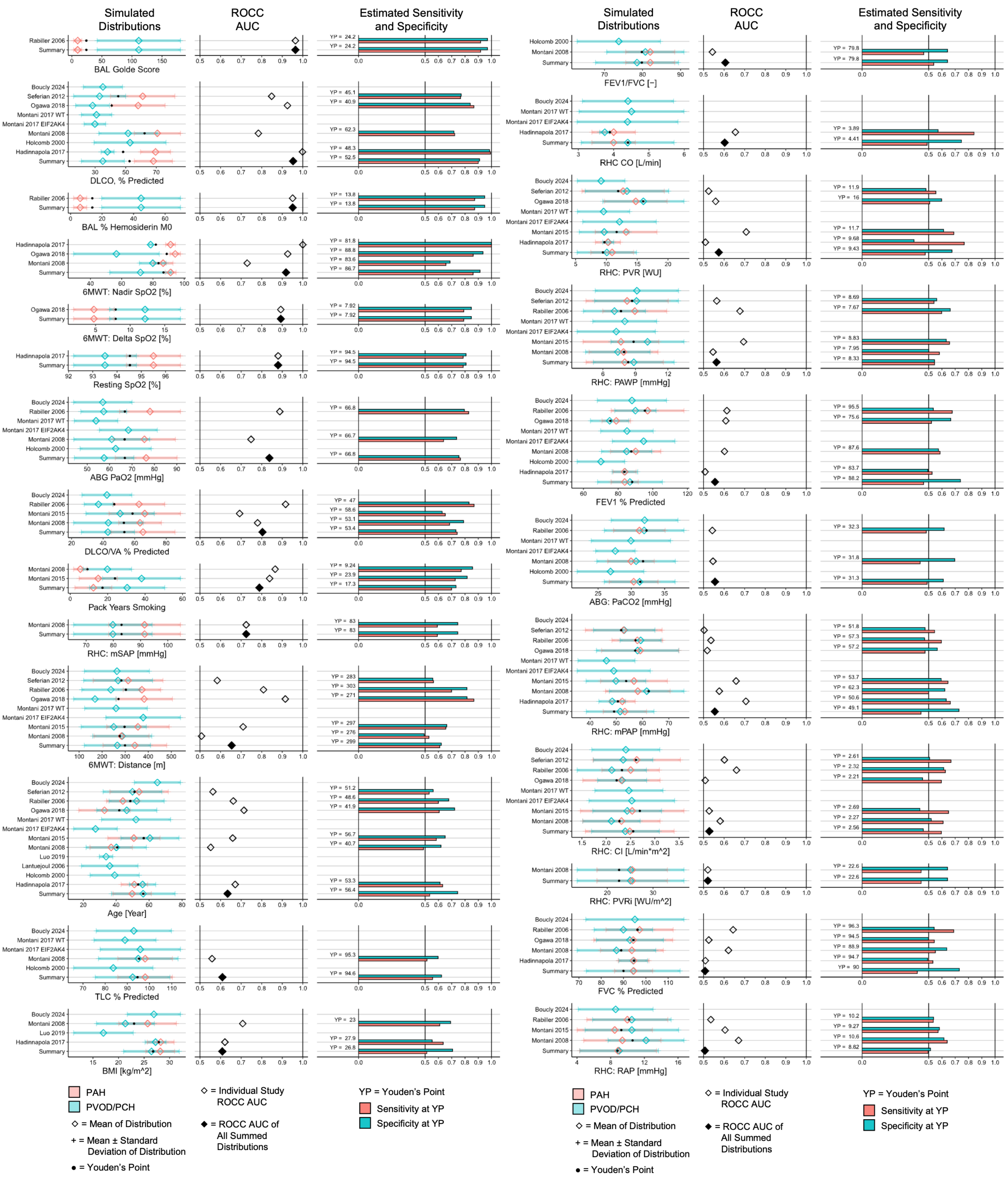

Supplemental Figure 2

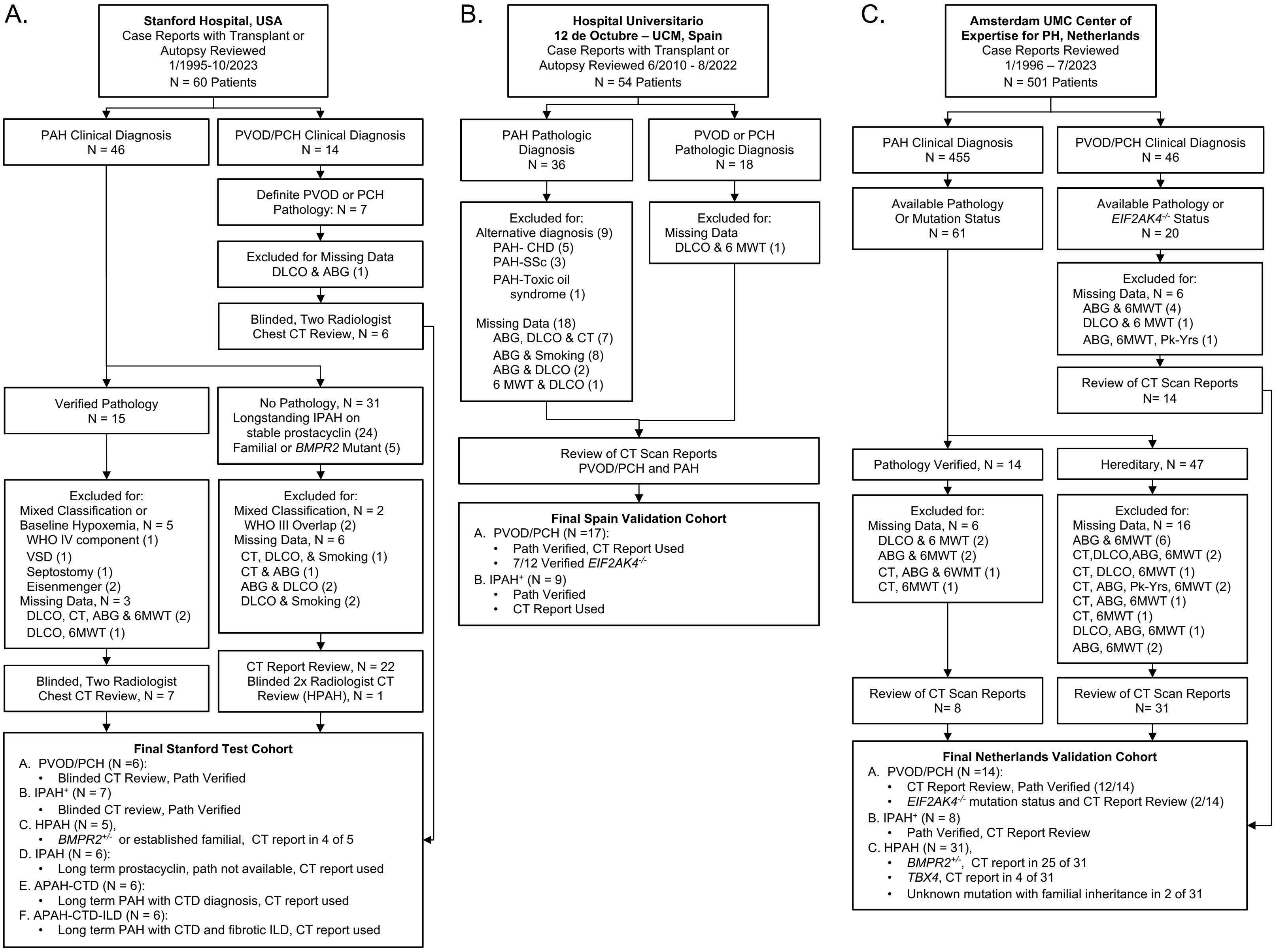

Supplemental Figure 3

Supplemental Table 2: Additional Stanford Baseline Characteristics							
	Classification:	IPAH		APAH: CTD		APAH: CTD-ILD	
	N:	6		6		6	
		μ (%)	σ	μ (%)	σ	μ (%)	σ
**RA**	mmHg	11	7	12	5	7	4
**mPAP**	mmHg	64	17	49	21	44	15
**CO**	L/min	4.3	1.4	3.2	0.8	3.4	1.0
**CI**	$L/min \cdot m^2$	2.2	0.6	2.0	0.5	1.9	0.5
**PVR**	WU	12.4	5.2	12.4	6.1	12.0	4.9
**DLCO**	% Predicted	0.83	0.14	0.75	0.23	0.49	0.12
**FVC**	% Predicted	0.89	0.19	0.73	0.22	0.76	0.23
**FEV1**	% Predicted	0.89	0.23	0.73	0.27	0.79	0.24
**FEV1/FVC**		0.90	0.10	0.92	0.17	0.92	0.12
**TLC**	% Predicted	0.78	0.21	0.80	0.21	0.71	0.07
**Never Smoker**		83%		83%		33%	
**Pack Years**		0.4	1.0	0.3	0.8	7.0	6.0
**BMI**	kg/m^2	31.3	8.5	25.7	6.7	25.6	4.2
**PaO2**	mmHg	84.6	5.5	85.4	19.5	83.3	11.1
**Nadir SpO2 6 MWT**	%	95	3.5	97	3.4	91	2.4
**GGO on CT**		33%		0%		17%	
**SLT on CT**		0%		0%		0%	
**LAD on CT**		0%		0%		50%	
**ILD on CT**		0%		0%		100%	
**Male**		33%		0%		0%	
**Age**	Years	37.2	11.7	40.2	16.4	50.8	11.2

Data presented as mean (μ) and standard deviation (σ) or percent (%)

Supplemental Table 2

Supplemental Table 3: Key PVOD/PCH Associated Conditions not Included in PVOD/PCH Likelihood Score

**Condition**	**Background**	**Reason For Exclusion from Likelihood Score and Example Publications**
Connective Tissue Disease (CTD)	Venous Remodeling is observed in 75-100% of in CTD-PAH patients.	A high incidence of venous remodeling in CTD patients suggests a sensitivity between 75 and 100% for PVOD/PCH in CTD-PAH. This wide range precludes an accurate estimate of sensitivity and specificity.<br><br>Dorfmuller et al. *Human Pathology* . 2007 38: 893-902<br>Overbeek et al. *ERJ* 2009 34: 371-379
Chemotherapeutic Agents	Alkylating agents (Mitomycin C, 5-Fluorouracil) have established associations with PVOD/PCH	37 Cases of chemotherapy related PVOD/PCH were identified in French Registry from 2004-2013. The number of PVOD/PCH cases in the French Registry is unknown, precluding calculating a sensitivity estimate. Our registries lack detailed exposure history for validation.<br><br>Ranchoux et al. *American Journal of Pathology* 2015 85: 356-371<br>Certain et al. *Chest* 2021 159:1197-1207
Occupational Exposure	Solvents and Trichloroethylene	72.7% of PVOD/PCH patients versus 27.7% of PAH patients in a case-control study had a significant exposure to organic solvents such as trichloroethylene. Median exposure time was 17 years with latency of 48 years. Therefore, a risk score is unlikely to outperform a careful occupational history.<br>Our registries lack detailed exposure history for validation.<br><br>Montani et al. *ERJ* 2015 46: 1721-1731
Hematopoietic Stem Cell Transplantation (hSCT)	Rare incidence of PVOD in younger patients undergoing hSCT.	Case series suggest hSCT-PVOD is a potentially reversible condition in the spectrum of graft versus host disease as opposed to fibrotic PVOD.<br><br>Bunte et al. *Bone Marrow Transplantation* 2008 41:677-686<br>Gunther et al. *American Journal of Pathology* 2019 189: 1159-1175
Sarcoidosis	Case Reports of Sarcoidosis related PVOD	Classified as WHO 5 PAH<br><br>Jones et al. *ERJ* . 2009 34: 517-520
Pulmonary Langerhans Cell Histiocytosis (PLCH)	Prominent venular involvement in PLCH	Classified as WHO 5 PAH<br><br>Fartoukh et al. *Am J Respir Crit Care Med.* 2000 161: 216-223
Sickle Cell Disease (SSc)	Case reports of SSc related PVOD	Classified as WHO 5 PAH<br><br>George et al. *Pulm Circ.* 2013 3: 952-958
Left Heart Disease	Known venous remodeling in mitral valve and other left heart disease.	Classified as WHO 2 PAH<br><br>Fayyaz et al. *Circulation.* 2018 17:1796-1810

**Supplemental Table 4: Inclusion Criteria if Missing Data**	
CT Available:	Two of Three variables from **A** (Lung Function/Oxygenation)<br>*and*<br>One of two variables from **C** (Patient Features)
CT Not Available:	Three of Three variables from **A** (Lung Function/Oxygenation)<br>*and*<br>Two of two variables from **C** (Patient Features)

Supplemental Table 4

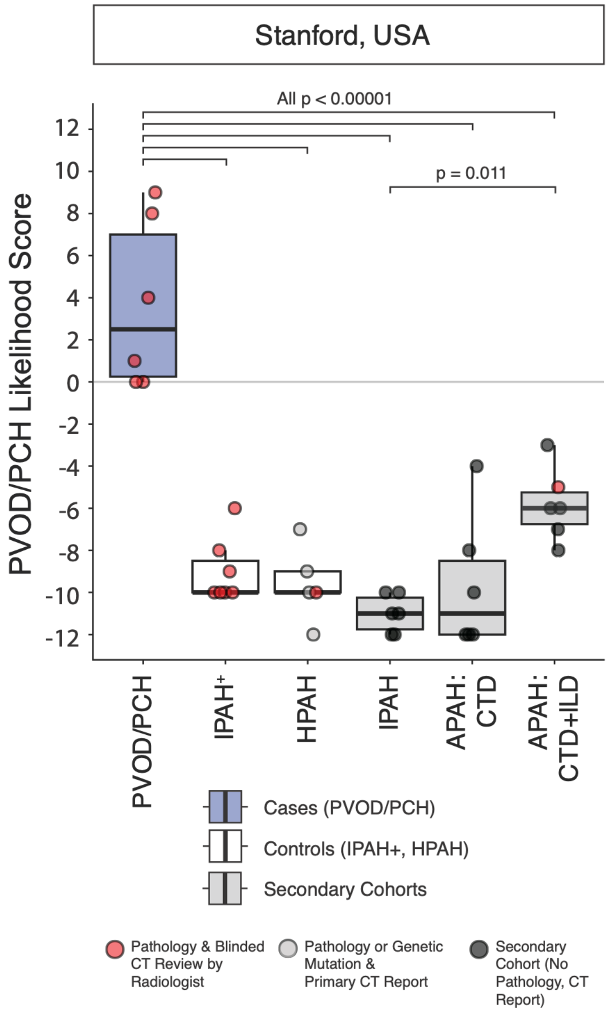

Supplemental Figure 4

Supplemental Table 5: Characteristics of Outlier Patients Designated in Figure 4

		Outlier 1	Outlier 2	Outlier 3
**Cohort**		**Spain**	**Spain**	**Netherlands**
**Class**		**PVOD**	**IPAH***	**HPAH**
**Missing Data**				Smoking Pk Yrs
**Mutation**				*BMPR2* $^{+/-}$
**RHC RA**	[mmHg]	4	10	14
**RHC mPAP**	[mmHg]	64	58	53
**RHC CO**	L/S	5.3	3.1	5.6
**RHC PVR**	WU	8	11	7.68
**DLCO**	% Predicted	76% (-2)	65% (0)	52% (0)
**FVC**	% Predicted	85%	85%	77%
**FEV1**	% Predicted	67%	72%	78%
**FEV1/FVC**		0.68	0.72	1.01
**Ever Smoker**		Yes	Yes	Yes
**Pack Years Smoking**		60 (0)	20 (0)	
**BMI**	kg/m^2	33	38	28
**ABG PaO2**	[mmHg]	65	61	64
**Lowest SpO2, 6 MWT**	%	90 (-2)	65 (+2)	
**Starting SpO2, 6 MWT**	%	90	90	
**GGO on Chest CT**		No (-1)	Yes (+1)	No (-1)
**SLT on Chest CT**		No (-2)	No (-2)	Yes (-2)
**LAD on Chest CT**		No (-2)	No (-2)	Yes (+2)
**Sex**		Female (0)	Female (0)	Male (+1)
**Age**	Years	33	38	58
**PVOD Likelihood Score**		-9	-1	0

Note green, red, and blue parentheses indicate points assigned based on the PVOD Likelihood score.

ABG = Arterial Blood Gas; BMI = Body Mass Index; CO = Cardiac Output; CT = Chest Computed Tomography Scan; DLCO = Diffusion Capacity for Carbon Monoxide; FEV1 = Forced Expiratory Volume in 1 Second; FVC = Forced Vital Capacity; GGO = Centrilobular Ground Glass Opacity; LAD = Lymphadenopathy; mPAP = Mean Pulmonary Artery Pressure; PaO2 = Arterial Partial Pressure of Oxygen; PVR = Pulmonary Vascular Resistance; RA = Right Atrial Pressure; RHC = Right Heart Catheterization; SLT = Septal Line Thickening; SpO2 = Peripheral Capillary Oxygen Saturation; WU = Wood Units; 6MWT = Six Minute Walk Test

A.

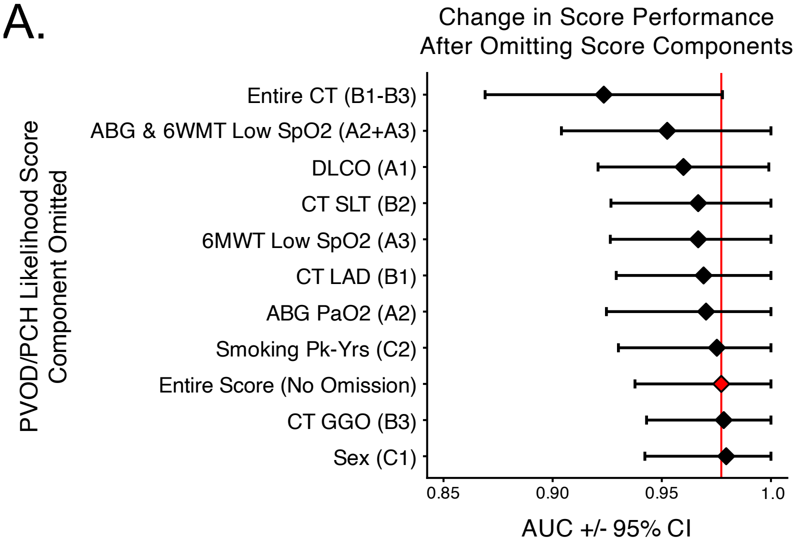

B.

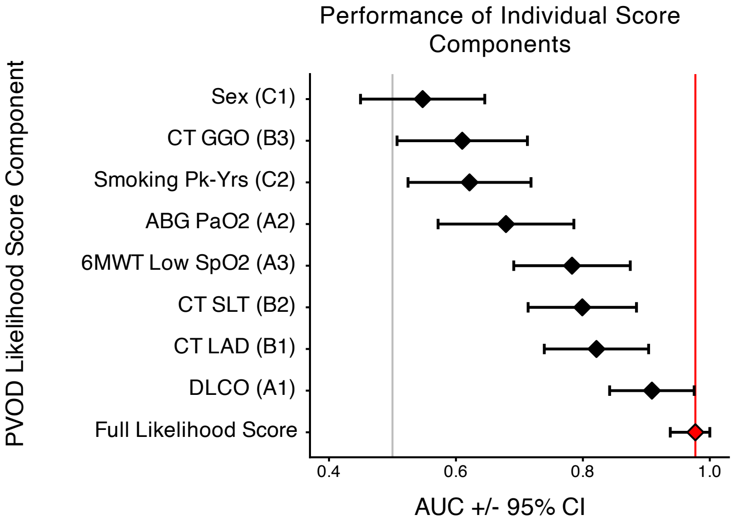

Supplemental Figure 5

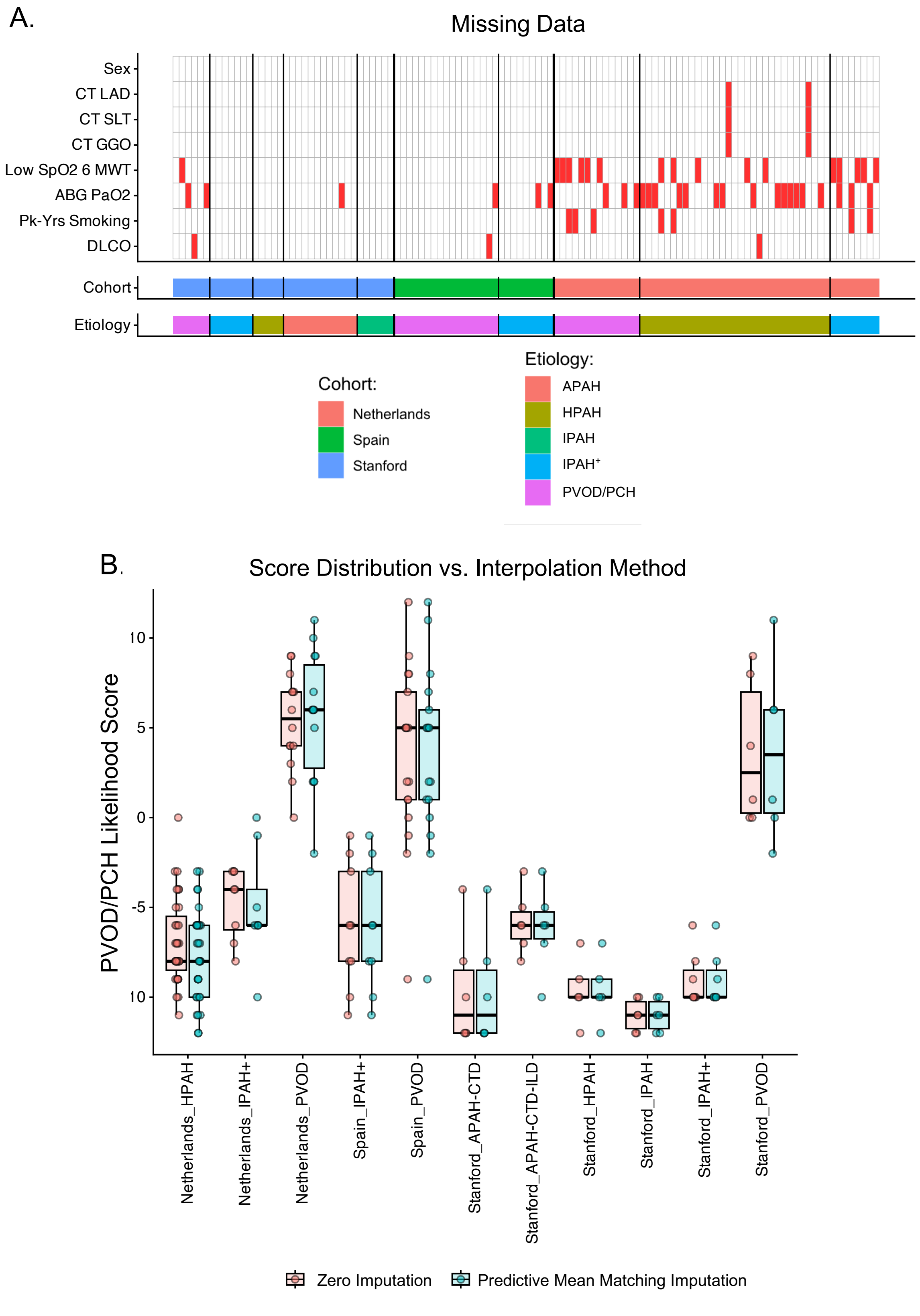

Supplemental Figure 6

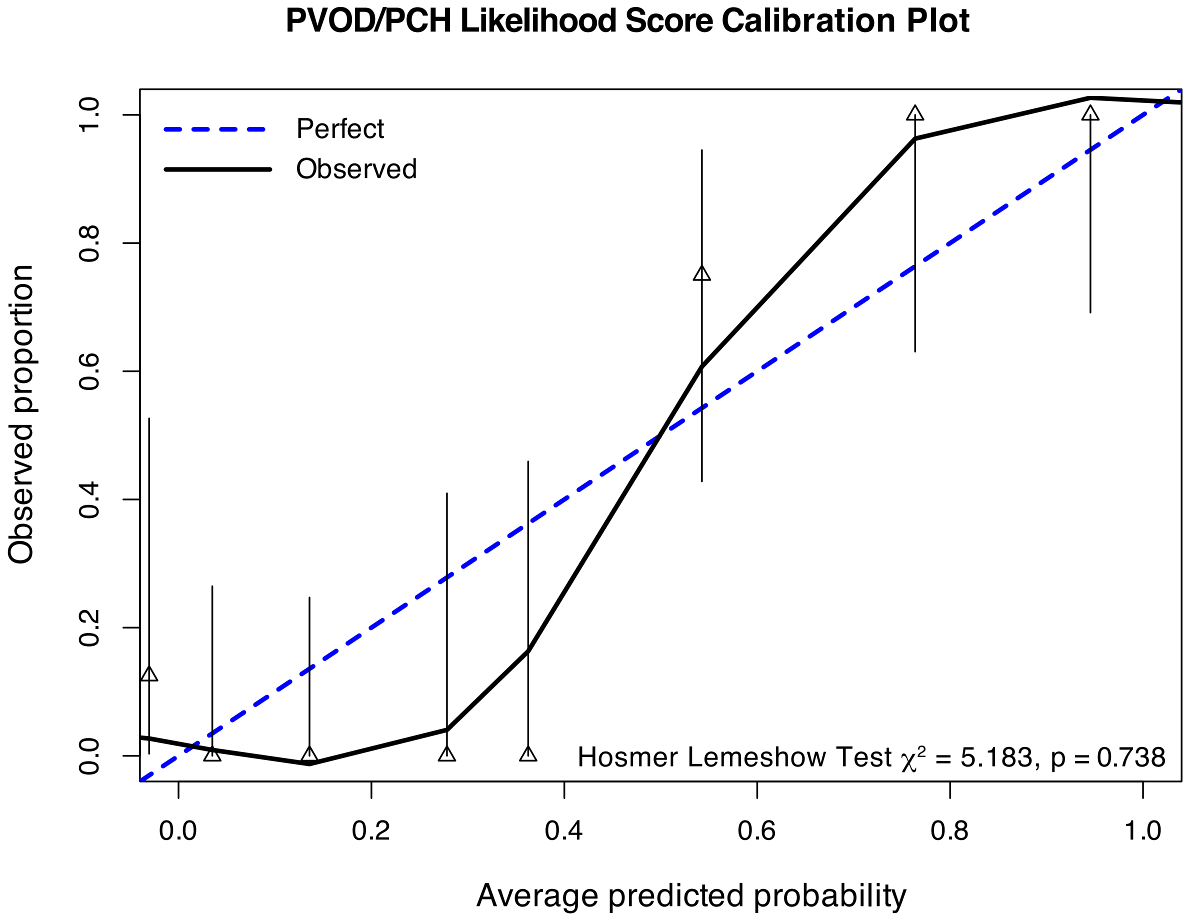

Supplemental Figure 7

Supplemental Table 6: Comparison of Different Pragmatic PVOD/PCH Likelihood Score Methods

Method	Details	ROC AUC (95% CI)	Score Range
A (Original)	For Sn or Sp > 80%, Weight +/- 2 Points<br>For Sn or Sp 70-79%, Weight +/- 1 Point	0.974 (0.930 - 1)	-12 to +12
B	For Sn or Sp >70%, Weight +/- 1 Point	0.969 (0.928 - 1)	-7 to +7
C	For Sn or Sp > 80%, Weight +/- 1 Point	0.977 (0.94 - 1)	-5 to +5
D	For Sn or Sp > 70%,<br>Weight by the Average of the Sn and Sp<br>(*e.g. 0.905 for DLCO, 0.89 for Six Minute Walk Test*)	0.974 (0.941 - 1)	-5.64 to +5.325

AUC = Area Under the Curve, DLCO = Diffusion Capacity of the Lung for Carbon Monoxide, ROC = Receiver Operator Characteristic, Sn = Sensitivity, Sp = Specificity

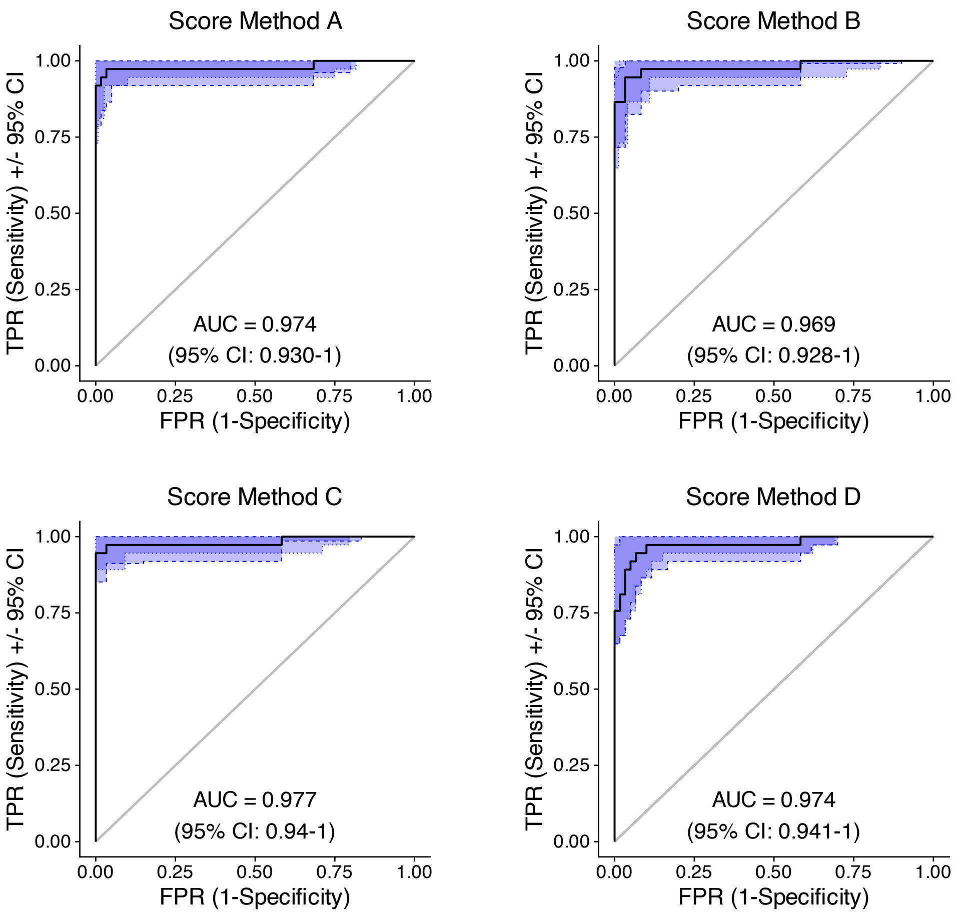

Supplemental Figure 8

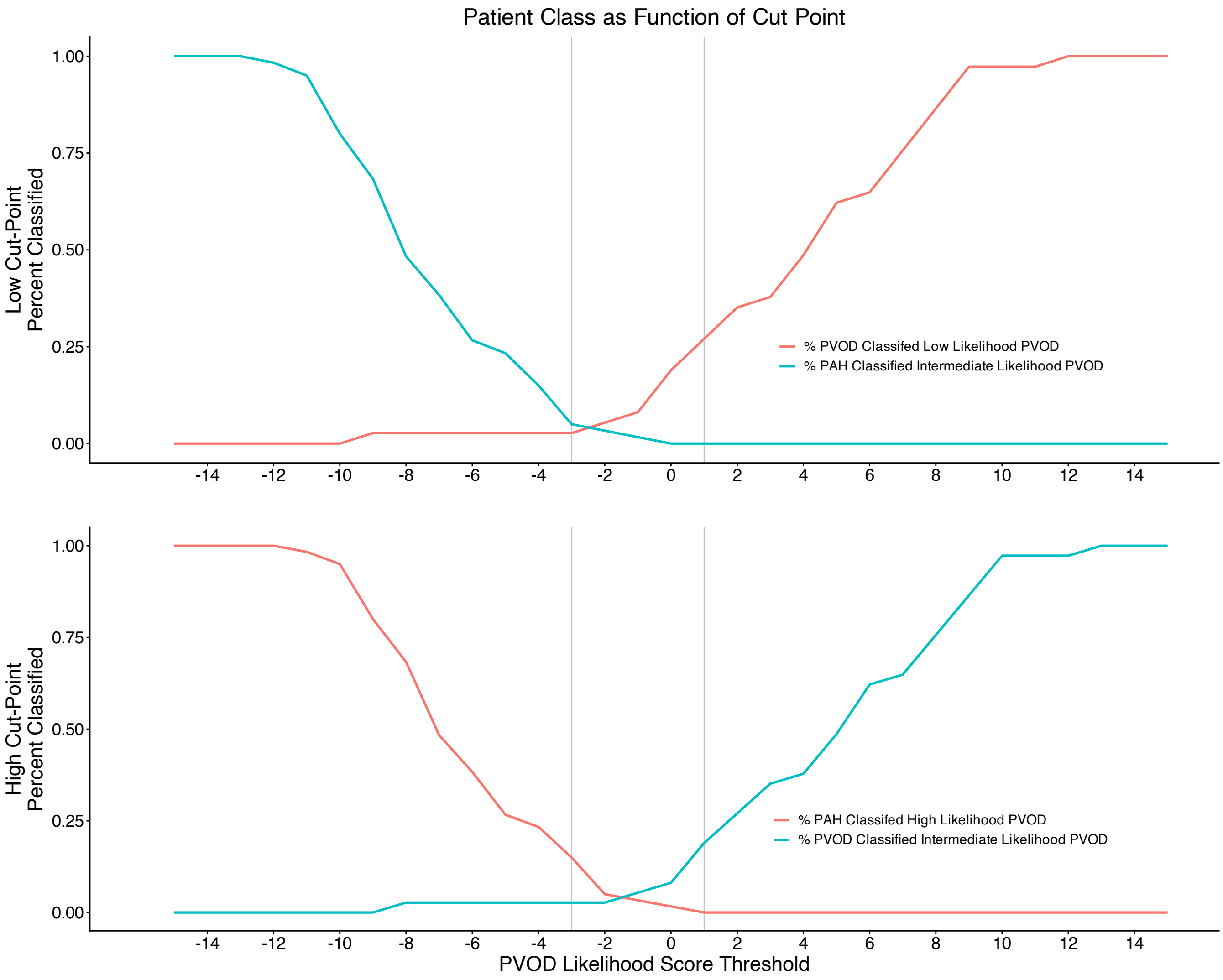

Supplemental Figure 9

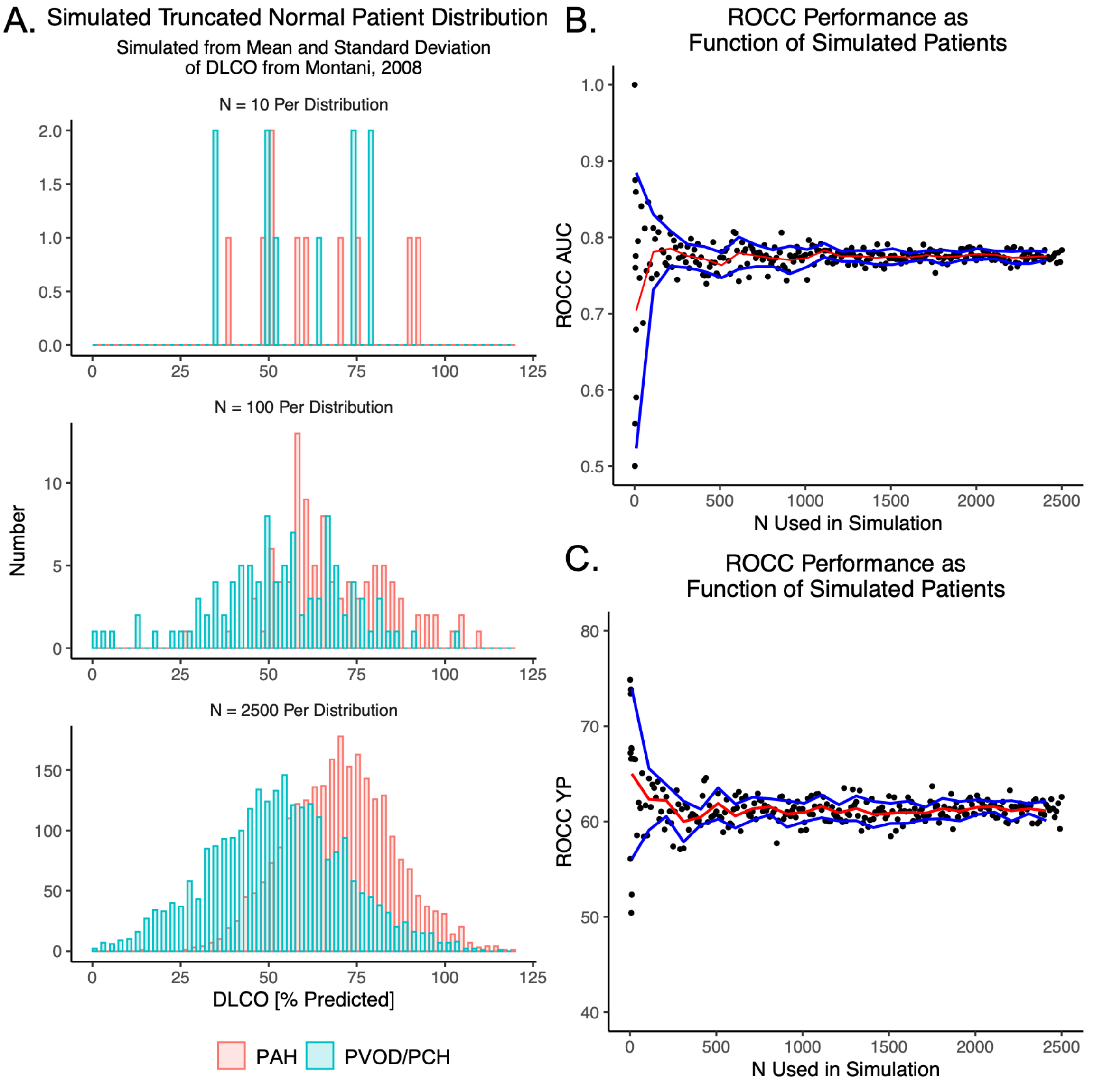

Supplemental Figure 10
